# Visualizing GABA transporters in vivo: an overview of reported radioligands and future directions

**DOI:** 10.1186/s13550-023-00992-5

**Published:** 2023-05-12

**Authors:** Niels Knippenberg, Matthias Bauwens, Olaf Schijns, Govert Hoogland, Alexandru Florea, Kim Rijkers, Thomas J. Cleij, Kasper Eersels, Bart van Grinsven, Hanne Diliën

**Affiliations:** 1 Sensor Engineering Department, Faculty of Science and Engineering, Maastricht University, 6200 MD Maastricht, The Netherlands; 2 Department of Nuclear Medicine, University Hospital Aachen, RWTH Aachen University, 52074 Aachen, Germany; 3 Department of Radiology and Nuclear Medicine, Maastricht University Medical Centre+ (MUMC+), 6229 HX Maastricht, The Netherlands; 4 Department of Neurosurgery, Maastricht University Medical Centre+ (MUMC+), 6229 HX Maastricht, The Netherlands; 5 School for Mental Health and Neuroscience (MHeNS), Maastricht University, 6200 MD Maastricht, The Netherlands; 6 Academic Center for Epileptology (ACE), Maastricht University Medical Centre+ (MUMC+), 6229 HX Maastricht, The Netherlands; 7 School for Cardiovascular Diseases (CARIM), Maastricht University Medical Centre+ (MUMC+), 6229 HX Maastricht, The Netherlands

**Keywords:** GABA, PET imaging, GABA transporter, GAT radioligand, GAT inhibitor

## Abstract

By clearing GABA from the synaptic cleft, GABA transporters (GATs) play an essential role in inhibitory neurotransmission. Consequently, in vivo visualization of GATs can be a valuable diagnostic tool and biomarker for various psychiatric and neurological disorders. Not surprisingly, in recent years several research attempts to develop a radioligand have been conducted, but so far none have led to suitable radioligands that allow imaging of GATs. Here, we provide an overview of the radioligands that were developed with a focus on GAT1, since this is the most abundant transporter and most of the research concerns this GAT subtype. Initially, we focus on the field of GAT1 inhibitors, after which we discuss the development of GAT1 radioligands based on these inhibitors. We hypothesize that the radioligands developed so far have been unsuccessful due to the zwitterionic nature of their nipecotic acid moiety. To overcome this problem, the use of non-classical GAT inhibitors as basis for GAT1 radioligands or the use of carboxylic acid bioisosteres may be considered. As the latter structural modification has already been used in the field of GAT1 inhibitors, this option seems particularly viable and could lead to the development of more successful GAT1 radioligands in the future.

**Supplementary Information:**

The online version contains supplementary material available at 10.1186/s13550-023-00992-5.

## Introduction

**Fig. 1:**
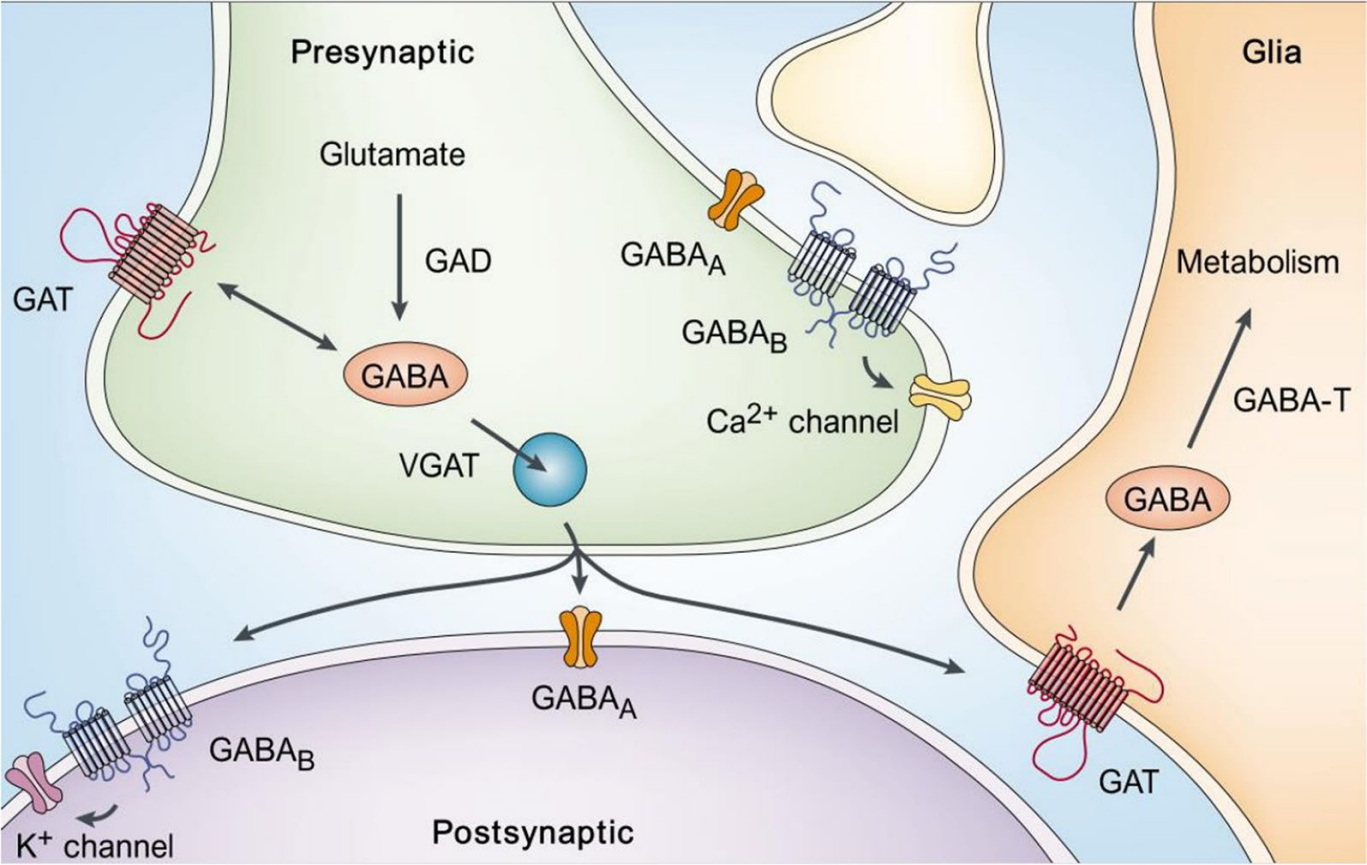
Schematic diagram of GABA synthesis and uptake. Reprinted with permission from Owens et al., *Nat. Rev. Neurosci.* 2002, *3* (9), 715–727. Copyright 2002 Springer Nature

The amino acid γ-aminobutyric acid (GABA (**1**)) is the main inhibitory neurotransmitter in the central nervous system (CNS). Synthesis of GABA occurs in GABA-producing neurons through the enzymatic decarboxylation of glutamate by two glutamate acid decarboxylase (GAD) enzymes (Fig. 1) [1]. Synthesized GABA is then stored into vesicles, into which it is transported by the vesicular GABA transporter (VGAT). GABA is released from these vesicles into the synaptic cleft by exocytosis. This process is regulated by voltage-dependent calcium channels, which allow calcium to pass into the presynaptic neuron upon depolarization. After exocytosis, GABA in the synaptic cleft can bind to the postsynaptic GABA_A_ and GABA_B_ receptors, which pass on the inhibitory signal to the postsynaptic neuron(s) [2, 3]. GABA signals are terminated by removal of GABA from the synaptic cleft by its reuptake into adjacent presynaptic neurons and glial cells by the GABA transporters (GATs).

A well-balanced status of inhibitory (e.g. GABA) and excitatory (e.g. glutamate) neurotransmission systems is required for a healthy brain function. Hence, disruptions in the GABAergic system could lead to an imbalance between the two neurotransmission systems and are associated with the pathogenesis of various CNS diseases, such as epilepsy, schizophrenia, Parkinson’s disease, and Alzheimer’s disease [4]. The GABAergic system is therefore a prime target of several CNS-targeted drugs [5], even though the exact role of GABA in these disorders is not fully understood. Non-invasive imaging of the GABAergic system could aid to understand this role.

**Fig. 2:**
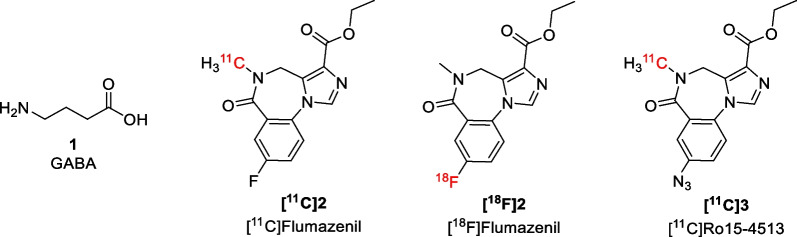
Structure of GABA and GABA_A_ radioligands

Several imaging methods have been developed for GABA receptors, with the benzodiazepine derivatives [^11^C]flumazenil (**[**^**11**^**C]2**), [^18^F]flumazenil (**[**^**18**^**F]2**), and [^11^C]Ro15-4513 (**[**^**11**^**C]3**) frequently being used as positron emission tomography (PET) tracers for the GABA_A_ receptor (Fig. 2) [5, 6]. For example, clinical studies using these radioligands include applications in schizophrenia, major depressive disorder, Alzheimer’s disease, and autism spectrum disorder [6]. Since the GABA receptors are mainly found on postsynaptic membranes, the GABA_A_ addressing tracers can provide information on postsynaptic GABA function. However, there are no PET tracers available yet to study presynaptic neuronal and glial GABAergic activity. Radiotracers for other presynaptic neuronal markers have been developed by addressing the neuronal and vesicular neurotransmitter transporters. For example, the use of PET tracers to localize and quantify dopamine transporters in patients with Parkinson’s disease is well established [7] and radioligands have also been developed for serotonin, noradrenalin, and glycine transporters [8]. However, there are no radioligands that can successfully image the GABA transporters (GATs) in vivo.

These GATs are membrane bound GABA/Na^+^ symporters, belonging to the solute carrier family SLC-6. As Na^+^/Cl^−^-dependent transporters, they entail the cotransport of two Na^+^ ions and one Cl^−^ ion [9–11]. The first GAT was isolated from rat brain by Radian et al*.* [12], after which this transporter was designated as GAT1. Following the isolation of GAT1 in rats, four different GAT subtypes have been cloned in various species leading to a complex nomenclature (Table 1) [13]. For the purpose of this review, the nomenclature proposed by the Human Genome Organization (HUGO) will be used (i.e. GAT1, BGT1, GAT2, and GAT3). It has been shown that the four GAT isoforms have a different distribution in the CNS [14]. GAT1—the most abundant transporter of the four GAT subtypes [15]—is mainly present on presynaptic GABAergic neurons, while GAT3 resides in astrocytes. Immunocytochemistry studies revealed that GAT2 is mainly located in the leptomeningeal cells, while BGT1 (betaine-GABA transporter 1) is present in the renal medullary cells.

**Table 1:** Overview of GABA transporter nomenclature for various species

Species	Nomenclature			
Mouse	mGAT1	mGAT2	mGAT3	mGAT4
Rat	rGAT1	rBGT1	rGAT2	rGAT3
Human	hGAT1	hBGT1	hGAT2	hGAT3
HUGO	GAT1	BGT1	GAT2	GAT3

Since the cellular distribution of the four GATs differs significantly depending on the isoform, it is preferable to develop selective GAT radioligands to suit the desired imaging application. As GAT1 is the most abundant and most of the research concerns this GAT subtype, this review will mainly focus on summarizing the efforts made to develop GAT1 addressing radioligands for in vivo imaging of presynaptic GABAergic neurons. The development of these radioligands is highly desirable, as they could contribute to our understanding of the pathogenesis of CNS disorders. This might be especially beneficial for schizophrenia and Parkinson’s disease, as in these disorders GATs have been shown to play an important pathophysiological role [4]. Several lines of evidence also suggest that patients with temporal lobe epilepsy have a lower GAT expression [16–20]. The recognition of GAT inhibitors to exhibit anticonvulsant properties then led to the development of the GAT1 inhibitor tiagabine (**11**, vide infra) [21–23], which is currently the only approved GAT1 inhibitor that is clinically used for the adjunctive treatment of epilepsy [24].

Several attempts have been made to develop GAT radioligands, which are summarized in this review. Since these attempts have mostly been unsuccessful, we specifically aim to elucidate why the radioligands that have been developed so far are of limited use. Based on our findings, we propose the use of non-nipecotic acid-based structures and the use of carboxylic acid bioisosterism as potential solutions for the successful development of GAT radioligands in the near future.

## Cyclic GABA analogues as inhibitors and radioligands

**Fig. 3:**
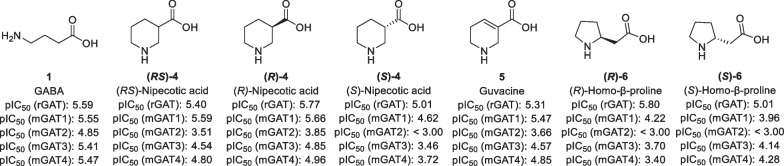
Structures of cyclic GABA analogues that have been used as GAT inhibitors. pIC_50_ values are obtained from rat brain slices [24, 25] or using cloned mGAT1-4 [27]

In order to develop GAT1 radioligands, a good understanding of small molecular weight GAT1 binders is of crucial importance. Given that a plethora of GAT1 inhibitors have already been developed, these molecules are a good starting point to develop GAT1 addressing radioligands. By the 1970s, it was known that cyclic analogues of GABA, such as nipecotic acid (**4**) and guvacine (**5**), can bind to the GABA binding site of GATs and function as GAT inhibitors (Fig. 3) [25, 26]. Further studies revealed that (*R*)-nipecotic acid ((*R*)-**4**) is about an order of magnitude more potent as GAT inhibitor than its enantiomer (*S*)-**4** [27, 28]. This difference was also found for homo-β-proline (**6**) [29]. Since these initial studies were conducted using rat brain slices, the results could not be specified for each of the GAT subtypes. However, the later cloning of the various GAT subtypes allowed for the determination of more specified IC_50_ values [30, 31], showing that these small amino acids mostly have a preference for GAT1.

**Fig. 4:**
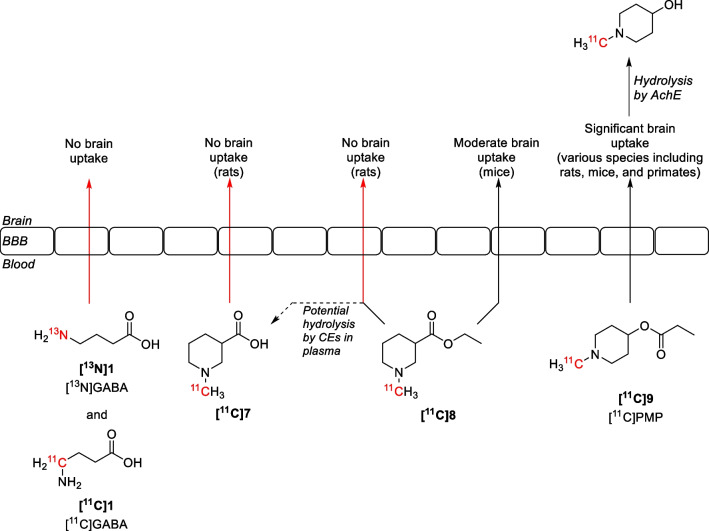
Schematic representation of the BBB permeability of small radiolabelled GABA analogues

Modelling studies showed that the amine and carboxylic acid functionalities of GABA and the above-mentioned cyclic GABA analogues are necessary for efficient binding into the GABA binding site of GATs [32–37]. However, these functionalities also give rise to zwitterionic behaviour, preventing these molecules from passing the blood–brain barrier (BBB) [38, 39]. Therefore, GABA and its (cyclic) analogues are of limited use in being a human biomarker. This was illustrated by early attempts to image the GABAergic system using ^13^N- and ^11^C-labelled GABA (**[**^**13**^**N]1** and **[**^**11**^**C]1**) (Fig. 4) [40, 41]. In later attempts, ^11^C-methylated nipecotic acid **[**^**11**^**C]7** was developed as potential GAT1 inhibitor, but also proved unsuccessful [42].

Because PET imaging studies in rats showed no brain uptake of **[**^**11**^**C]7** due to the reasons discussed above, the ester intermediate **[**^**11**^**C]8** was tested. However, this molecule did not cross the rat’s BBB either. This result is more surprising, as previous imaging studies from 1998 using mice show a moderate uptake of **[**^**11**^**C]8** in the brain (2.5–5.5% injected dose per gram of tissue (ID/g)) [43]. Moreover, **[**^**11**^**C]8** is both structurally and chemically related to *N*-[^11^C]methylpiperidin-4-ylpropionate ([^11^C]PMP, **[**^**11**^**C]9**), which is a radiotracer used in PET imaging of acetylcholinesterase (AchE) [44, 45]. A potential explanation for the latter issue could be that **[**^**11**^**C]8** and **[**^**11**^**C]9** are hydrolysed by different esterases due to the different attachment of the ester functionality. Previous research indicates indeed that **[**^**11**^**C]8** is not hydrolysed by AchE, but by other carboxylesterases (CEs) [43]. A different expression of these esterases in plasma could then lead to the hydrolysis of **[**^**11**^**C]8** before brain entry. This would be consistent with the suspected hydrolysis of **[**^**18**^**F]39** in rats (vide infra), though further research would be required to find a definite answer.

## Lipophilic GABA analogues as inhibitors and radioligands

The failed trials to image GATs using small analogues of GABA introduce a second requirement for a successful radioligand besides docking into the GABA binding site: BBB permeability. While several PET tracers, such as 6-[^18^F]fluoro-L-DOPA and [^18^F]FDG, are able to cross the BBB through carrier-mediated transport [46], nipecotic acid-related compounds are not known to be transported in such a way. Therefore, transcellular diffusion seems the most feasible way to make GAT1 radioligands cross the BBB. Fortunately, several strategies to optimize key physiochemical parameters in order to enhance membrane diffusion have been developed in medicinal chemistry. Lipinski’s Rule of Five, which relates membrane permeability to molecular weight, lipophilicity, and hydrogen bonding [47], is one of the best known examples [48]. Adaptations and extensions of the Rule of Five for CNS drugs in specific have also been made, in which the existing limits were refined and more properties were added [49–52]. A common theme for these strategies is to improve the lipophilicity, which has also been done in the field of GAT inhibitors and radioligands.

### Lipophilic *N*-substituted GAT1 inhibitors

**Fig. 5:**
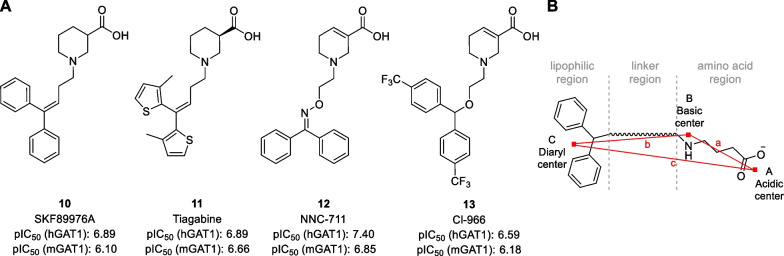
**A** Structures of GAT1 inhibitors SKF89976A **10**, tiagabine **11**, NNC-711 **12**, and Cl-966 **13**. pIC_50_ values are obtained using cloned hGAT1 [57] or mGAT1 [27]. **B** Graphical representation of the pharmacophore model of GAT inhibitors

For the field of GAT inhibitors, the BBB problem was solved by the addition of a lipophilic moiety to the small amino acids described above. This lipophilic moiety most often has the form of an *N*-alkyl spacer connected to a biaryl system. Such a system was first reported by Ali et al*.*, who synthesized various *N*-(4,4-diphenyl-3-butynyl)amino acid derivatives [28]. The resulting compounds, such as SKF89976A **10**, were not only more lipophilic, but also more potent GAT inhibitors than their parent amino acids (Fig. 5A). Following the original report, several other research groups have synthesized similar derivatives. For example, bioisosteric replacement of the phenyl rings of **10** by 2-thienyl moieties gave rise to tiagabine **11** [21–23], which is currently the only approved drug targeting GAT1. Other well-known GAT1 inhibitors include the guvacine analogues NNC-711 **12** [53] and Cl-966 **13** [54] exhibiting an oxime and ether spacer. All these compounds are more potent as GAT1 inhibitor than their parent amino acids (compare Fig. 5A and Fig. 3 for mGAT1). Modelling studies suggest that further interactions between the lipophilic tail and hydrophobic regions of the GAT could give rise to this increased potency [35, 36, 55]. Modelling studies also allowed for the development and validation of a pharmacophore model of lipophilic GAT inhibitors (Fig. 5B) [56]. This pharmacophore model includes three features: an amino acid region with the acidic centre A and the basic centre B and a lipophilic region with the diaryl centre C, which is connected by a linker. By investigating several known GAT inhibitors, the distances between the pharmacophore features were found to be within the following range: *a* = 3.9–5.6 Å, *b* = 3.8–7.8 Å, *c* = 3.4–9.7 Å, and ∠*ABC* = 42°–147°

**Fig. 6:**
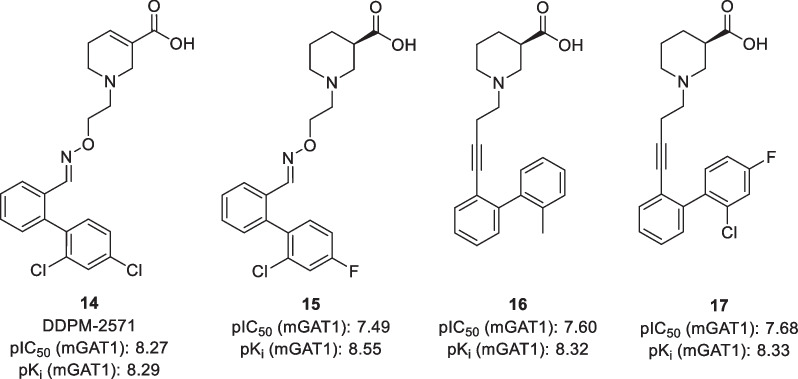
Structures of selected GAT1 inhibitors. pIC_50_ and pK_i_ values are obtained using cloned mGAT1 [59, 61, 62]

Based on the success of these second-generation GAT1 inhibitors, further selective and potent inhibitors have been developed over the last two decades [57–59]. An overview of these lipophilic GAT1 inhibitors is presented in Additional file 1: Tables S1–S7. Wanner and co-workers published several studies and, to the best of our knowledge, developed the most potent GAT1 inhibitor to date. Their compound, DDPM-2571 **14**, is an NNC-711 derivative that was found after the screening of oxime libraries using MS binding assays (Fig. 6) [60]. In vivo studies showed that this inhibitor was effective in the prevention of induced seizures in mouse models [61], though no further in vivo testing has been performed afterwards. Compound **15**, the GAT1 inhibitor with the highest affinity, is part of an analogous series of nipecotic acid derivatives that has been synthesized by the same research group [62]. The group of Wanner has also developed potent GAT1 inhibitors with carbon linkers, for which they found that inhibitors with alkyne linkers and a biphenyl moiety have the highest potency [63]. Optimization of the linker revealed that a C_4_ linker has the optimal length and that compounds with an alkyne linker have a higher potency than analogous inhibitors with an alkene linker [64]. Substitution of the terminal aryl group then afforded compounds such as **16** and **17** with potencies in the same range than those of the above-mentioned oxime series without this potentially labile functionality.

### Synthesis of lipophilic GAT1 radioligands

**Scheme 1:**
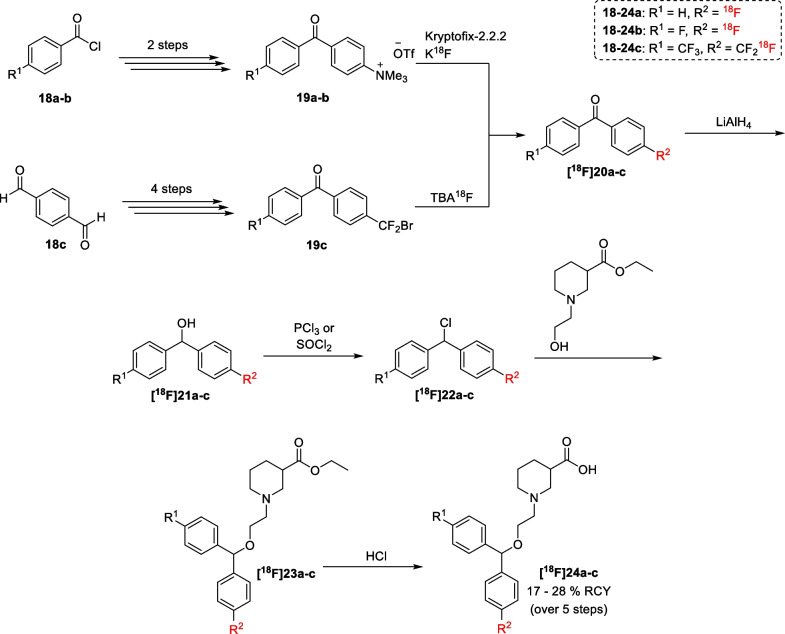
Radiosynthesis of compounds **[**^**18**^**F]24a-c**

Using the more efficient lipophilic GAT1 inhibitors as starting point, several attempts have been made to synthesize a viable GAT1 radioligand based on these inhibitors. While the biological evaluations of these radioligands are summarized in the section "Biological evaluation of GAT radioligands", their synthesis is outlined in the next two sections. The first radioligands, radiolabelled Cl-966 derivatives **[**^**18**^**F]24a-c**, were synthesized by Kilbourn et al*.* in 1990 through a rather lengthy radiosynthesis (Scheme 1) [65]. For radioligands **[**^**18**^**F]24a-b**, the radiosynthesis started from the aryltrimethylammonium triflate precursors **19a-b**, which were obtained from the acyl chlorides **18a-b** [66, 67]. On the other hand, the synthesis of compound **[**^**18**^**F]24c** was started from brominated precursor **19c**. Following nucleophilic fluorination, a reduction and chlorination were performed to access intermediates **[**^**18**^**F]22a-c**. These intermediates were subsequently reacted with the ethyl ester of *N*-(2-hydroxylethyl)nipecotic acid to afford the radioligands **[**^**18**^**F]24a-c** after deprotection of the ester functionality.

**Scheme 2:**
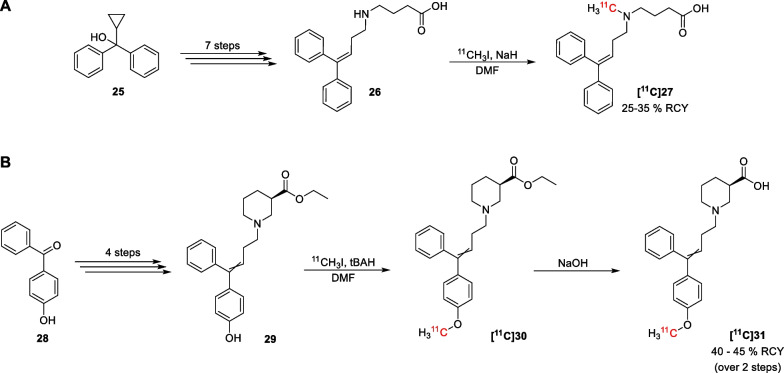
Radiosynthesis of compounds **[**^**11**^**C]27** (**A**) and **[**^**11**^**C]31** (**B**)

A few years later Le Bars et al*.* reported the synthesis of the ^11^C-labelled lipophilic GABA derivative **[**^**11**^**C]27** [68], based on their promising results using the non-labelled derivative as GABA uptake inhibitor [69] (Scheme 2A). The radiolabelled analogue was obtained through methylation of *N*-diphenylbutenyl GABA **26**, for which the synthesis has been reported by Ali et al*.* [28]. Another ^11^C-labelled radioligand has been reported by Vandersteene et al*.* [70]. Their compound **[**^**11**^**C]31** is a [^11^C]methoxy-labelled analogue of the GAT1 inhibitor SKF89976A. Starting from 4-hydroxybenzophenone (**28**), the phenol precursor **29** was obtained in four steps (Scheme 2B) [71]. The radioligand **[**^**11**^**C]31** was then synthesized through a methylation reaction using [^11^C]methyl iodide followed by deprotection of the ester functionality in alkaline conditions.

**Scheme 3:**
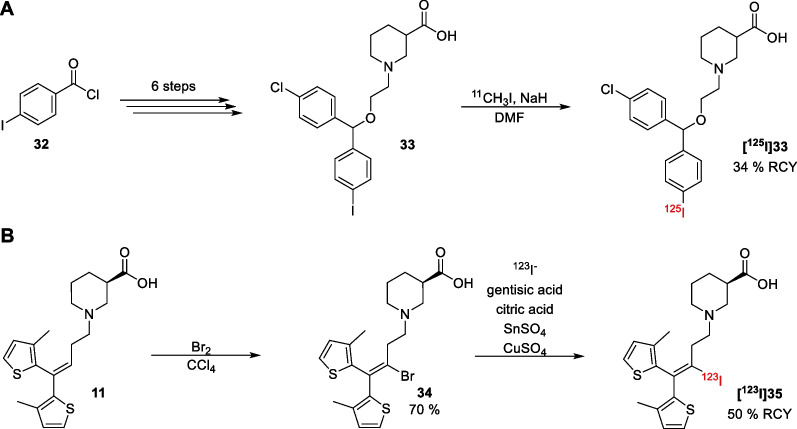
Radiosynthesis of compounds **[**^**125**^**I]33** (**A**) and **[**^**123**^**I]35** (**B**)

Furthermore, ^125^I-labelled CIPCA **[**^**125**^**I]33** has been synthesized by Van Dort et al*.* (Scheme 3A) [72]. In their approach, CIPCA **33** was obtained from 4-iodobenzoyl chloride (**32**) in six steps. Afterwards, **[**^**125**^**I]33** was synthesized through a solid state isotopic exchange in a 34% radiochemical yield (RCY). In a more recent trial, tiagabine **11** was successfully labelled with ^123^I by Schijns et al*.* [73]. In this synthesis, tiagabine was brominated to give radiolabelling precursor **34** in a 70% yield (Scheme 3B). Through a Cu(I)-assisted halogen exchange, the radiolabelled derivative **[**^**123**^**I]35** was then obtained in 50% RCY.

**Scheme 4:**
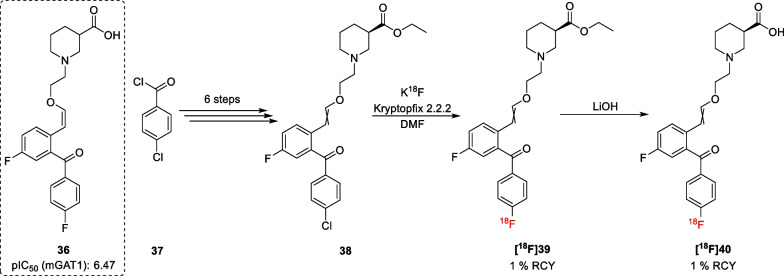
Radiosynthesis of compound **[**^**18**^**F]40**

Lastly, Sowa et al*.* developed the radioligand **[**^**18**^**F]40** [42], which was inspired by a series of GAT1 inhibitors developed by Quandt et al*.* [74]. These GAT1 inhibitors exhibit an asymmetrical bis-aromatic residue connected to the nipecotic acid core through a vinyl ether spacer. Optimization of the methanone-bridged compounds showed that the (*Z*)-isomer was slightly more potent. Moreover, it was found that the addition of fluorine substituents increased the potency and selectivity with respect to the non-substituted derivative, leading to compound **36** as the most potent inhibitor of this series (Scheme 4). Given the electron deficient aromatic system, the radiolabelled derivative **[**^**18**^**F]39** could be accessed through a nucleophilic aromatic substitution from the chlorinated precursor **38**. Further deprotection of the ester functionality afforded the radioligand **[**^**18**^**F]40**.

### Synthesis of radioligands for other GAT subtypes

**Fig. 7:**
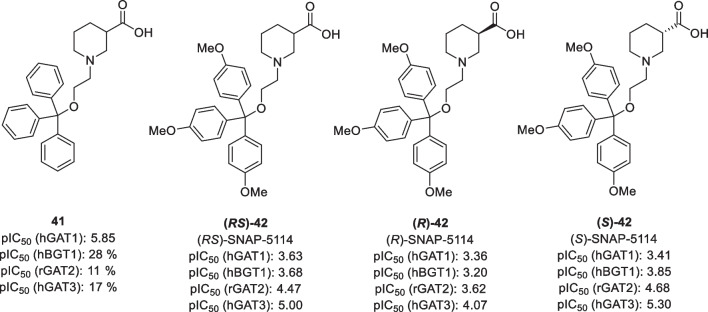
Structures of selected GAT3 inhibitors. pIC_50_ values are obtained using cloned GATs [26]. Percentages indicate the per cent inhibition at 100 μm

**Scheme 5:**
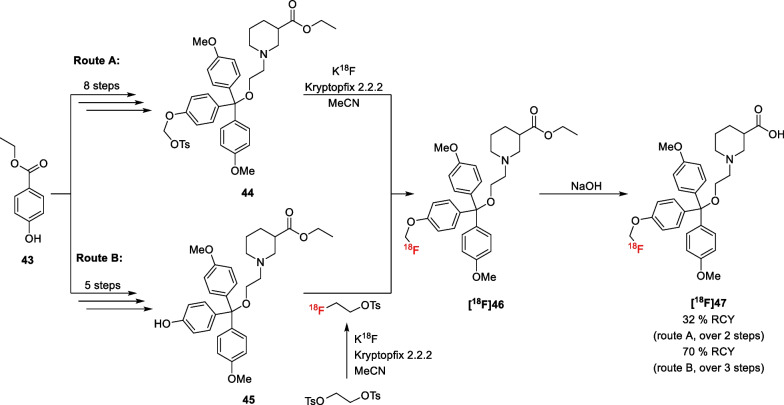
Radiosynthesis of compound **[**^**18**^**F]47**

As discussed earlier, efforts to develop GAT radioligands have focussed on GAT1, mainly due to its high abundancy among the GAT subtypes and its presynaptic cellular distribution. In contrast, no attempts to develop a radioligand for BGT1 and GAT2 have been reported to date. For GAT3, however, Schirrmacher et al*.* attempted to synthesize a radioligand which they based on GAT3 inhibitor (*S*)-SNAP-5114 **(S)-42** [75]. This inhibitor was developed by Dhar et al*.*, who found that the addition of a third aryl moiety to the lipophilic GAT1 inhibitors causes selectivity for GAT3 (Fig. 7) [30]. While the non-substituted trityl derivative **41** was more potent for GAT1, introduction of methoxy substituents on the para positions increased the affinity for GAT3. Further studies into the stereochemical preferences led to compound (*S*)-SNAP-5114 **(S)-42**. The radiolabelled derivative [^18^F]fluoroethyl SNAP-5114 **[**^**18**^**F]47** was first accessed from the tosylate precursor **44**. However, the synthesis of this precursor from compound **43** and subsequent labelling proved to be difficult (i.e. route A, Scheme 5) and a different approach using 2-[^18^F]fluoroethyltosylate was developed (route B). In this procedure, precursor **45** was reacted with separately synthesized [^18^F]fluoroethyltosylate to give ester intermediate **[**^**18**^**F]46**. Subsequent hydrolysis of the ester protecting group afforded radioligand **[**^**18**^**F]47** in 70% RCY.

### Biological evaluation of GAT radioligands

**Fig. 8:**
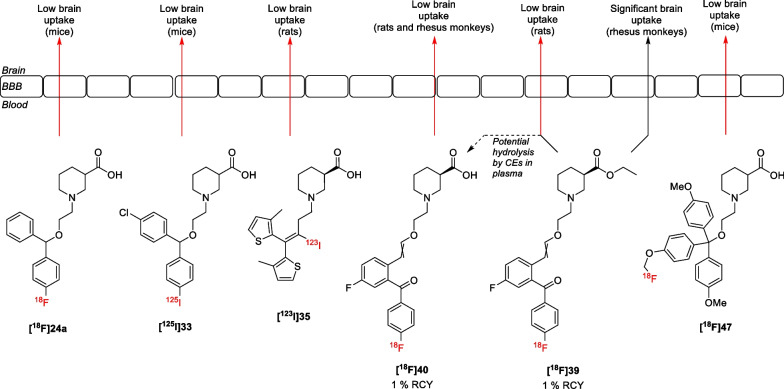
Schematic representation of the BBB permeability of lipophilic GAT radioligands

For most of the reported GAT radioligands, in vivo imaging studies have also been conducted (Fig. 8). Preliminary results of the first reported radioligand, **[**^**18**^**F]24a**, indicated that the compound exhibited low brain permeability in mice [65]. Despite the low brain uptake, a heterogeneous brain distribution (i.e. cortex/striatum ratio of 1.44) was obtained, which is similar to [^3^H]tiagabine [76]. However, no further studies were done to optimize these radioligands due to, among other reasons, the found toxicity of Cl-966 [42, 77, 78]. Similar results were obtained for the structurally related ^125^I-labelled CIPCA **[**^**125**^**I]33**. Although a ^123^I-labelled derivative would be needed for clinical single-photon emission computerized tomography (SPECT) imaging, studies using **[**^**125**^**I]33** still provided useful information on the brain uptake of GAT radioligands. Imaging studies in mice showed a low brain uptake of the radioligand (i.e. 0.82% of the injected dose) [72]. Thyroid radioactivity concentrations showed < 1% in vivo deiodination, ruling out this option as cause for the low brain uptake. A slight heterogeneous distribution (i.e. cortex/striatum ratio of 1.2) was obtained, which is significantly lower than the [^3^H]tiagabine ratio [76]. This might be one of the reasons why no further studies to synthesize the (*R*)-isomer or the ^123^I-labelled analogue were conducted. Besides the mediocre results of the early GAT1 radioligands, the GAT3 radioligand **[**^**18**^**F]47** also suffered from low brain uptake (0.3% ID/g) during preliminary in vivo imaging studies in mice [75].

**Fig. 9:**
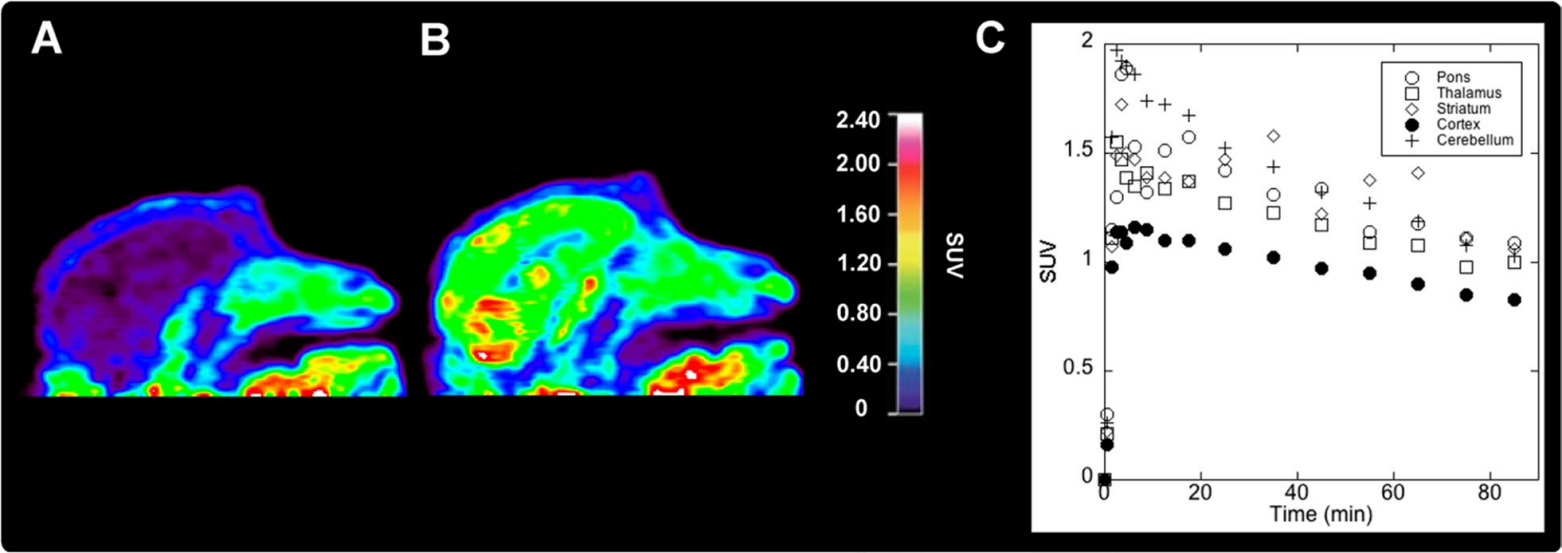
Baseline non-human primate imaging with **[**^**18**^**F]40** (**A**), **[**^**18**^**F]39** (**B**), and time radioactivity curves for **[**^**18**^**F]39** (**C**). Reprinted with permission from Sowa et al., *ACS Chem. Neurosci.* 2018, 9, 2767–2773. Copyright 2018 American Chemical Society

More recently, radiolabelled iodotiagabine **[**^**123**^**I]35** has been synthesized. In vivo gamma camera whole-body images in rodents showed that the radioligand appeared in the head, suggesting it had passed the BBB [20]. However, more detailed SPECT images proved that the radioligand was present in the nasal mucosa or Harderian glands instead of the brain. The authors suggest that altered biophysical properties due to the addition of the iodine or possible deiodination could explain why the radioligand failed to enter the brain. Moreover, radioligand **[**^**18**^**F]40** was also shown to exhibit poor BBB permeability during initial PET imaging studies in rodents [42]. Repeated imaging following pretreatment with the P-glycoprotein (Pgp) inhibitor cyclosporine A showed no difference in brain uptake, indicating that the radioligand **[**^**18**^**F]40** is not a substrate for the Pgp efflux transporter. Further studies using the ester **[**^**18**^**F]39** were then undertaken in order to verify whether the carboxylic acid moiety limits the BBB permeability of **[**^**18**^**F]40**. However, these experiments did not show brain uptake of **[**^**18**^**F]39** either. In contrast to the results in rats, PET imaging studies in rhesus monkeys using **[**^**18**^**F]39** and **[**^**18**^**F]40** showed significant brain uptake of the ester **[**^**18**^**F]39** in the cortex, thalamus, striatum, and cerebellum (i.e. standardized uptake value (SUV) ≈ 1 after 90 min; Fig. 9). The authors suggest that differences in esterase expression could lead to the hydrolysis of **[**^**18**^**F]39** in rats before brain entry, which would explain the different uptake of **[**^**18**^**F]39** between the species.

Despite the brain uptake of ester **[**^**18**^**F]39**, it is unlikely that the radioligand has specific affinity for GAT1, as the free carboxylic acid group of the nipecotic acid moiety is essential for specific binding. Several studies have shown that nipecotic acid ester prodrugs have no affinity for GAT1 and require in situ hydrolysis in order to selectively bind to GAT1 [79–82]. Alternatively, the radioligand **[**^**18**^**F]39** could behave like a prodrug and the ester could hydrolyse after crossing the BBB to give the carboxylic acid **[**^**18**^**F]44** that binds to GAT1. While such prodrugs have been used for preclinical PET imaging [83, 84], they complicate quantitative analysis and kinetic modelling of imaging data. Hence, the radioligand **[**^**18**^**F]39** is not suitable for the in vivo imaging of neuronal GATs.

## Alternatives for nipecotic acid-based structures

The insufficient brain uptake of the nipecotic acid-related radioligands discussed above and the significant brain uptake of ester **[**^**18**^**F]43** seem to suggest that the unprotected nipecotic acid moiety hinders the BBB permeability. Given that several brain-penetrating PET tracers with free carboxylic acid moieties have been reported [85, 86], the presence of the carboxylic acid does not necessarily disqualify a molecule from passing the BBB. Rather, it seems that the zwitterionic nature of the nipecotic acid moiety causes these problems.

As shown above, the addition of lipophilic substituents to the nipecotic acid residue increased the BBB permeability and allowed for the development of GAT1 inhibitors (e.g. tiagabine) that show sufficient brain build-up to achieve a therapeutic effect. However, the BBB permeability of lipophilic radioligands is still insufficient to visualize GATs in vivo. These findings are also in line with pharmacological research of tiagabine, which suggests that tiagabine might exhibit a slow equilibration between plasma and brain [87, 88]. While this is less of an issue for therapeutic drugs, it can thwart the rapid brain uptake required for imaging purposes. Altogether, the above observations support the idea that the direct radiolabelling of GAT1 inhibitors might not be the optimal strategy and alternatives for such nipecotic acid-based structures might need to be developed.

### Non-classical GAT1 inhibitors

**Fig. 10:**
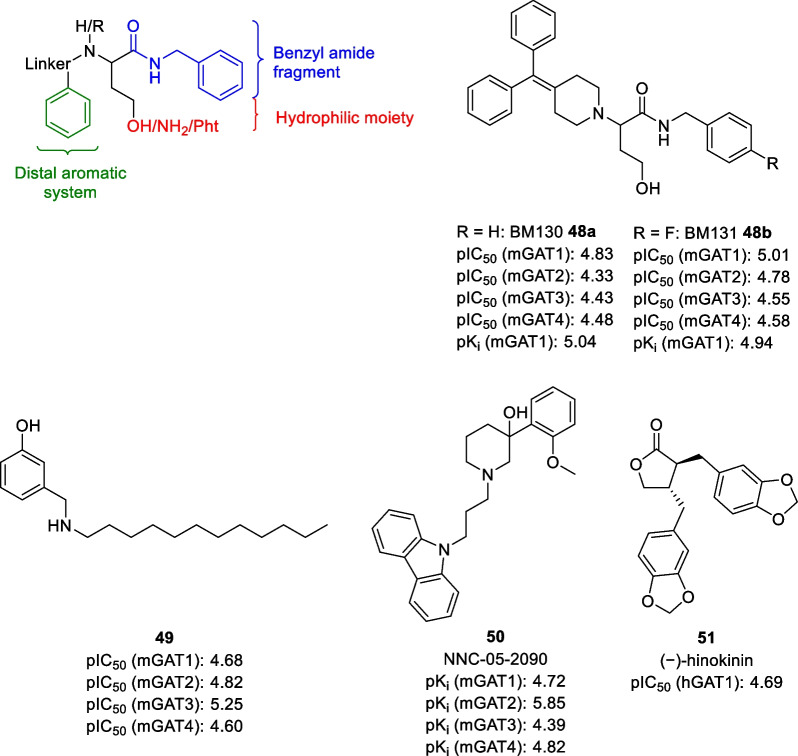
Pharmacophore model of 2-substituted 4-hydroxybutanamides and structures of selected non-classical GAT inhibitors. pIC_50_ and pK_i_ values are obtained using cloned mGAT1-4 or hGAT1 [90, 94–96]

A potential solution to circumvent the above-mentioned problem would be to use non-classical GAT inhibitors that are not based on nipecotic acid or similar amino acids as a basis for developing GAT1 radioligands. Although less models and structure activity relationships have been developed for such non-classical GAT inhibitors, a few classes of compounds have been explored. For 2-substituted 4-hydroxybutanamides, it was found that a benzyl substituent on the amide group, a distal aromatic substituent at the 2-position, and a hydrophilic moiety at the 4-position are crucial for their activity (Fig. 10) [89, 90]. However, after screening compounds with various linkers, aromatic systems, and different hydrophilic functionalities at the 4-position (alcohols, amines, and phthalimides) no selective GAT inhibitors were found within this class of compounds [90–93]. Most of the active inhibitors display a broad inhibitory profile for all four GAT subtypes instead. For example, compounds BM130 and BM131 **48a-b** are the most promising mGAT1 inhibitors within this series, but also inhibit mGAT2-4.

Similar problems were observed for aminomethylphenols, which have also been evaluated as GAT inhibitors. *N*-alkylated derivatives like **49** were already reported in the early 1980s and found to inhibit neuronal GABA uptake and glial β-alanine uptake in vitro [94]. Full GAT subtype selectivity was determined in 2008 by Kragler et al*.*, who found that inhibitor **49** has a broad inhibitory effect for all four GAT subtypes with a slight preference for mGAT3 [95]. Unfortunately, variations in the lipophilic moiety and the position of the hydroxyl group only had small effects, showing that it is difficult to develop selective GAT inhibitors within this class of compounds.

Another class of GAT inhibitors worth mentioning are the 4-methoxyphenylpiperidin-4-ol derivatives, which exhibit a modified nipecotic acid residue. However, this modification seems to eliminate the high affinity for GAT1, giving inhibitors that are mainly selective for GAT2. NNC-05-2090 **50** is the most potent GAT2 inhibitor of this series and exhibits a more than tenfold selectivity over other GATs [96]. Another non-conventional GAT inhibitor has been found by Timple et al*.*, who showed that the lignan(−)-hinokinin (**51**) acts as a non-competitive inhibitor of hGAT1 [97]. Unfortunately, this compound is not selective for GAT1 either, as it was found to inhibit dopamine and the norepinephrine transporters as well.

As can be observed from the above overview, modifications or omissions of the nipecotic acid residue seem to result in GAT inhibitors that either display a broad inhibitory profile for all GAT subtypes or are otherwise not selective for GAT. Therefore, major developments would be necessary to develop a non-nipecotic acid-based GAT1 inhibitor, which makes it complicated to use such non-classical GAT inhibitors as basis for the development of selective GAT1 radioligands.

### Bioisosteres

Besides the use of non-classical GAT1 inhibitors as basis for GAT1 selective radioligands, another solution to overcome the zwitterionic nature of the nipecotic acid moiety would be to use carboxylic acid bioisosteres. This potential solution has also been proposed by Sowa et al*.* [42]. Fortunately, several carboxylic acid bioisosteres have been developed and are frequently applied in medicinal chemistry to create structural derivatives with similar biological properties [98–100]. Several of these bioisosteres have also been applied to GABA and its analogues.

**Fig. 11:**
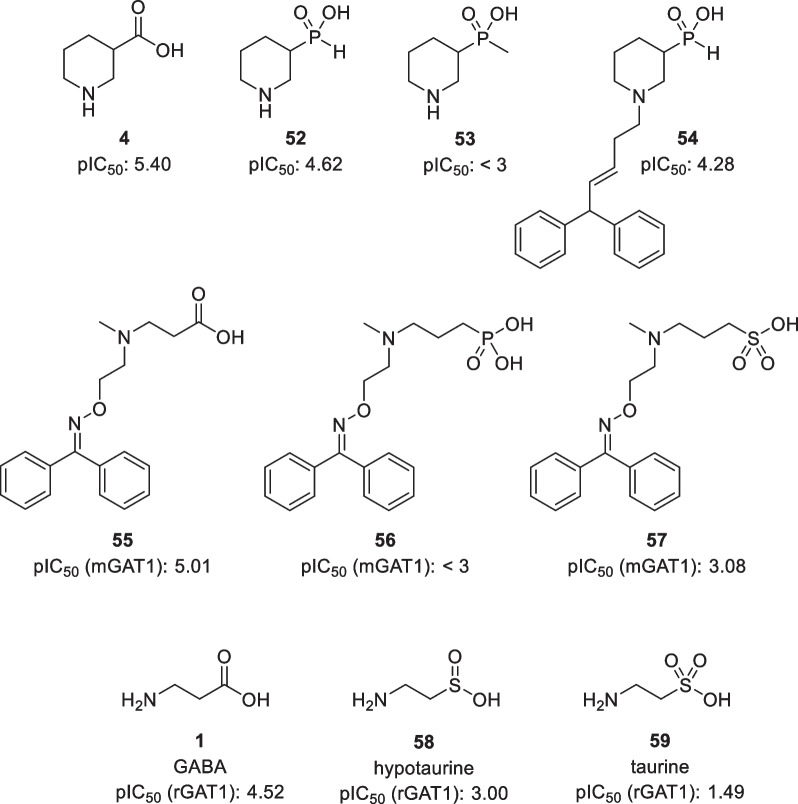
Structure of phosphinic, phosphonic, sulphinic, and sulphonic acid derivatives of GABA and nipecotic acid. pIC_50_ values are obtained from rat brain synaptosomes (top row) [101] or from cloned mGAT1 [102] or rGAT1 [14] (middle and bottom row, respectively)

For example, Kehler et al*.* synthesized phoshinic acid derivatives of nipecotic acid and tested those in [^3^H]GABA uptake assays (Fig. 11) [101]. It was found that phosphinic acid **52** shows a moderate potency about tenfold weaker than nipecotic acid. On the other hand, the methylphospinic acid derivative **53** completely killed the activity. Interestingly, introduction of the lipophilic *N*-(4,4-diphenyl-3-butenyl) group to afford **54** did not lead to an increased potency. Lipophilic phosphonic acid and sulphonic acid analogues of GABA **56** and **57** also did not show any activity for GAT1 [102], although this might also be due to the additional carbon in the structure after replacement of the carboxylic acid. Moreover, hypotaurine **58** and taurine **59** exhibiting the sulphinic and sulphonic acid functionalities were shown to have no to minimal affinity to GAT1 [14, 103]. Therefore, these functional groups do not seem to be a viable bioisostere for GAT inhibitors.

**Fig. 12:**
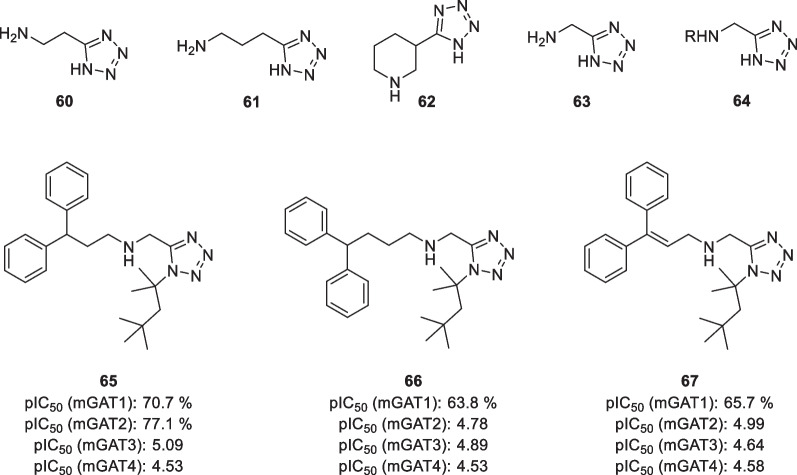
Structure of tetrazole derivatives of several amino acids. pIC_50_ values are obtained from cloned mGAT1-4 [105]. Percentages represent specific binding remaining in the presence of 100 μm inhibitor

Moreover, tetrazoles have been explored as potential carboxylic acid bioisosteres (Fig. 12). As early as 1984, Schlewer et al*.* synthesized several tetrazole amino acids [104]. Inhibition of GABA uptake was tested for derivatives of β-alanine, GABA, and nipecotic acid **60–62** in rat brain synaptosomes, but neither of them showed promising potencies (pIC_50_ < 4) [105]. More recently, Schaffert et al*.* have presented several lipophilic tetrazole analogues of glycine in a search for novel mGAT1-mGAT4 inhibitors [105]. Their parent structure **63** showed no activity in any of the four GAT subtypes, which is similar to glycine. Interestingly, the addition of lipophilic residues to give monosubstituted lipophilic derivatives **64** did not enhance the potency. 1,5-Disubstituted tetrazole derivatives also showed only marginal inhibition for mGAT1, although several compounds were found to act as moderate inhibitors for mGAT2-4. For example, diphenylpropyl derivative **65** showed moderate inhibition for mGAT3 and mGAT4. Lengthening of the alkyl chain or introduction of a double bond to give **66** and **67** also resulted in an inhibitory effect in mGAT2.

**Fig. 13:**
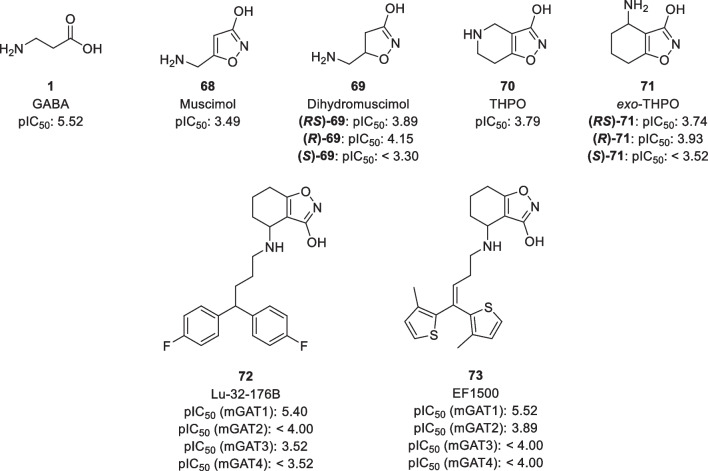
Structure of muscimol and THPO derivatives. pIC_50_ values are obtained from rat brain synaptosomes [107, 110] (top row) or from cloned mGAT1-4 [111] (bottom row)

Muscimol **68** is another bioisosteric analogue of GABA with a 3-isoxazolol moiety replacing the carboxylic acid [106]. While muscimol and direct analogues such as 4,5-dihydromuscimol **69** show moderate effects as GABA uptake inhibitors (Fig. 13) [25, 107], they are also potent agonists of the ionotropic GABA receptors [108, 109]. Therefore, these analogues are of limited use in developing selective GAT1 radioligands. Further development using muscimol as a lead compound led to THPO **70** and derivatives as selective GABA uptake inhibitors after incorporating the amino sidechain into the ring [106]. While substitution of the 3-isoxazolol moiety back to a carboxylic acid functionality afforded the potent GAT inhibitor nipecotic acid, moving the amino group of THPO to an exocyclic position as in **71** was less effective [110]. Nevertheless, several lipophilic *exo*-THPO analogues have been synthesized exhibiting a 3-hydroxyisoxazol moiety as bioisosteric replacement for the carboxylic acid functionality [111, 112]. Despite several of them being selective GAT1 inhibitors (e.g. Lu-32-176B **72** and EF1500 **73**), the *exo*-THPO moiety has also been shown to be zwitterionic and exhibits a low BBB permeability [110].

**Scheme 6:**
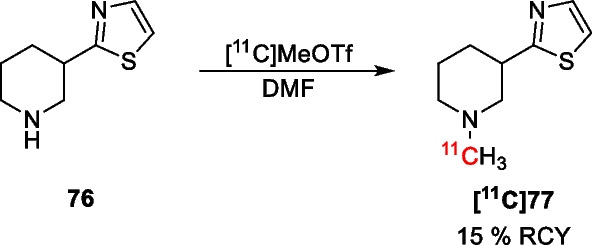
Synthesis of radioligand **[**^**11**^**C]77**

Further studies regarding carboxylic acid bioisosterism in GAT inhibitors have been reported by Sowa, who performed an exploratory [^3^H]GABA uptake inhibition assay for several nipecotic acid bioisosteres [113]. These preliminary results (Table 2) show that ethyl nipecotate **74** and THPO **70** are of limited use as bioisosteric replacements due to their low potency. However, in contrast to earlier results, the tetrazole **62** showed promising inhibition of GABA uptake. Unfortunately, attempts to synthesize this tetrazole derivative were met with problems as no satisfactory separation of the tetrazole and the 3-cyanopiperidine starting material could be obtained, making further investigation of this bioisostere difficult. Instead, further focus was devoted to using thiazole **76** as bioisosteric replacement. In order to allow in vivo imaging, this bioisostere was radiolabelled using [^11^C]MeOTf, to give radioligand **[**^**11**^**C]77** (Scheme 6). PET imaging studies using this tracer in rats gave excellent brain uptake with a maximum SUV of 4. Similar results were obtained in rhesus monkeys, in which a maximum whole brain SUV of 3 was obtained. Further analysis showed that radioligand was mostly taken up in the striatum (maximum SUV ≈ 4).

**Table 2:** Results of the preliminary screening of nipecotic acid bioisosteres as GAT inhibitors by Sowa [113]

Compound	Inhibition (%)	Normalized to tiagabine
	− 3	− 10.7
	− 4	− 14.3
	4	14.3
	9	32.1
	− 6	− 21.4
Tiagabine	28	100

Inhibition was measured using [^3^H]GABA uptake inhibition assay in rat brain homogenate

## Outlook and conclusion

The studies summarized in this review demonstrate that most attempts to create GAT radioligands have been unsuccessful up until now, mostly due to insufficient brain uptake. This low brain uptake is proposed to be caused by the zwitterionic nature of the nipecotic acid moiety. Developing GAT1 radioligands without this nipecotic acid moiety is difficult, because it facilitates binding into GAT1. Hence, no selective non-classical GAT1 inhibitors are available to use as a basis for GAT1 radioligands. Therefore, further efforts should focus on developing strategies to increase the brain permeability of nipecotic acid-based compounds.

While several PET tracers, such as 6-[^18^F]fluoro-L-DOPA and 2-[^18^F]FDG, are able to cross the BBB through carrier-mediated transport [46], nipecotic acid-related compounds are not known to be transported in such a way. Hence, like most current PET imaging agents, passive diffusion seems the most feasible way for GAT1 radioligands to enter the brain [49]. In order to facilitate this diffusion, lipophilic moieties have been attached to nipecotic acid to access tiagabine and derivatives. However, radiolabelled analogues of these lipophilic GAT1 inhibitors still show insufficient brain uptake in order to be useful human biomarkers. The uptake of ester **[**^**18**^**F]39** showed that the incorporation of a masked carboxylic acid moiety could be a viable strategy. These masked carboxylic acids are used in two strategies: prodrugs and bioisosteres. While prodrugs are less ideal due to difficult quantitative analysis and kinetic modelling, the use of carboxylic acid bioisosteres seems to be promising strategy. After all, a variety of carboxylic acid bioisosteres have been developed and as visible in the above overview have precedent in the field of GAT inhibitors. Moreover, it has been shown that thiazole **[**^**11**^**C]76** exhibits excellent brain uptake, indicating that these bioisosteric replacements can improve the BBB permeability significantly.

**Fig. 14:**
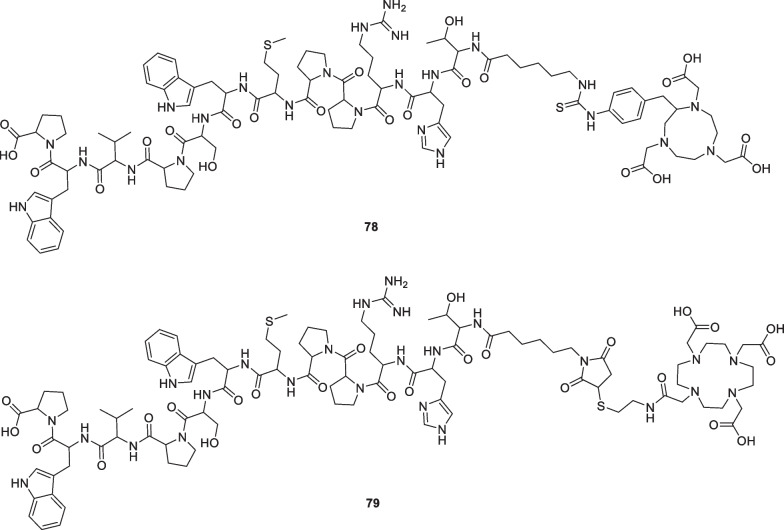
Structure of labelled transferrin receptor targeting peptides **78** and **79**

Less explored options to increase BBB permeability could include disruption of the BBB in order to increase the paracellular diffusion of radioligands [114, 115]. However, there are only limited studies available that use this approach to increase the BBB permeability of PET tracers. Nevertheless, several studies have shown promising results [116]. For example, BBB disruption using focussed ultrasound significantly increased the brain uptake of [^18^F]2-fluoro-2-deoxy-sorbitol [117]. Besides BBB disruption, linking the radioligand to a carrier system could also enable transport across the BBB by exploiting natural transport mechanisms [46]. Also for this option, limited studies on PET tracers have been conducted, making it difficult to achieve a fast application in the field of GAT radioligands. Pioneering studies synthesized several ^18^F and ^68^Ga-labelled transferrin receptor targeting peptides in order to evaluate their potential to actively transport small molecular weight compounds through the BBB (Fig. 14) [118]. Due to a difficult radiosynthesis and purification of the ^18^F-labelled analogues, only the ^68^Ga-labelled NOTA and DOTA derivatives **[**^**68**^**Ga]78** and **[**^**68**^**Ga]79** were used for further experiments. In vitro cell uptake experiments showed that both peptides exhibit negligible cellular uptake. Moreover, in vivo experiments using the DOTA derivative showed an extremely low brain uptake of the peptide **[**^**68**^**Ga]79**, indicating that further development is necessary to efficiently use these carrier systems to increase the BBB permeability of PET tracers. The same can be said for nanoparticles, which have also been recognized as promising carrier systems for brain delivery of medicine and nuclear probes [119, 120]. For example, studies showed that nanoparticles can be used as carrier agents to deliver molecular imaging dyes across the BBB for MRI applications [121]. Moreover, several efforts have been performed in the radiolabelling of nanoparticles in order to access nanoparticle PET tracers [122, 123], which could serve as precedent to apply this technology for the development of brain-permeable GAT1 radioligands.

Given the little precedent of applying BBB disruption and carrier systems in order to develop brain-permeable PET tracers, there are still major challenges that need to be resolved. For example, in the case of carrier systems the potential loss of binding affinity of the imaging agents is a remaining risk. Therefore, the use of BBB disruption or carrier systems could work as a long-term solution in order to improve the BBB permeability of the GAT1 radioligands. The use of carboxylic acid bioisosteres could lead to a faster solution given the more extensive use of these masked carboxylic acids in the field of GAT1 inhibitors.

Taken together, the proposed strategies to increase the BBB permeability in combination with the increased knowledge on small molecular weight binders for GAT1 could lead to the development of more successful GAT1 radioligands in the future.

## Supplementary Information

**Additional file 1.** Overview of lipophilic *N*-substituted nipecotic acid and guvacine based GAT1 inhibitors.

## Data Availability

Not applicable.

## Abbreviations

AchE: Acetylcholinesterase
BBB: Blood–brain barrier
BGT1: Betaine-GABA transporter 1
CE: Carboxylesterase
CNS: Central nervous system
GABA: γ-Aminobutyric acid
GAD: Glutamate acid decarboxylase
GAT: GABA transporter
HUGO: Human Genome Organization
PET: Positron emission tomography
Pgp: P-glycoprotein
RCY: Radiochemical yield
SPECT: Single-photon emission computerized tomography
SUV: Standardized uptake value
VGAT: Vesicular GABA transporter
