# Supplementary material for: Visualizing GABA transporters in vivo: an overview of reported radioligands and future directions
Source: EJNMMI Res. 2023 May 12;13:42. doi: 10.1186/s13550-023-00992-5 (PMC10182260; doi:10.1186/s13550-023-00992-5)
Supplement: Supplementary file 1 — Additional file 1. Overview of lipophilic N-substituted nipecotic acid and guvacine based GAT1 inhibitors. [file 13550_2023_992_MOESM1_ESM.docx]

**Additional file 1**

**Visualizing GABA transporters in vivo: an overview of reported radioligands and future directions**

Niels Knippenberg, Matthias Bauwens, Olaf Schijns, Govert Hoogland, Alexandru Florea, Kim Rijkers, Thomas J. Cleij, Kasper Eersels, Bart van Grinsven, Hanne Diliën

**Overview of lipophilic *N*-substituted nipecotic acid and guvacine based GAT1 inhibitors 2**

Table S1: SAR of GAT1 inhibitors with an ether linker 2

Table S2: SAR of GAT1 inhibitors with a vinyl ether linker 5

Table S3: SAR of GAT1 inhibitors with an oxime linker 7

Table S4: SAR of GAT1 inhibitors with an amine linker 14

Table S5: SAR of GAT1 inhibitors with an alkene linker 17

Table S6: SAR of GAT1 inhibitors with an alkene and allene linker 24

Table S7: SAR of GAT1 inhibitors with an alkyne linker 26

**References 27**

| ***Table S1:*** *SAR of GAT1 inhibitors with an ether linker* | | | | | | | | | | | | | | | | | | | | | | |  |  |  |  |
| --- | --- | --- | --- | --- | --- | --- | --- | --- | --- | --- | --- | --- | --- | --- | --- | --- | --- | --- | --- | --- | --- | --- | --- | --- | --- | --- |
|  |  | |  | | **1a** | | **1b** | | | **1c** | | | **1d** | | **1e** | | **1f** | | **1g** | | **1h** | | | | | |
|  |  | |  | |  | | | | |  | | |  | |  | |  | | | |  | | | | | |
| **Entry** | **R_1_** | | **R_2_** | | **Nip pIC_50_^a^** | | **Guv**  **pIC_50_ ^a^** | | | **pIC_50_ ^b^** | | | **pIC_50_^b^** | | **pIC_50_ ^b^** | | **Nip**  **pIC_50_ ^b^** | | **Guv**  **pIC_50_^b^** | | **pIC_50_ ^b^** | | | | | |
| **a** | Ph | | Ph | | 5.86 ^[1],b^  5.80 ^[2],b^  5.47 ^[3]^  5.52 ^[4]^  *Propyl:*  4.96 ^[4]^ | |  | | | 5.24 ^[5, 6]^  *Meta derivative:*  *5.25 ^[6]^* | | | 6.90 ^[7]^ | | 6.48 ^[8, 9]^ | | 7.26 ^[10]^  7.29 ^[11]^ | | 7.32 ^[10]^  7.31 ^[11]^ | | 6.95 ^[8, 9]^ | | | | | |
| **b** | Ph | | H | | < 5 ^[12],b^ | |  | | | 4.19 *^[6]^*  *Butyl derivative:*  *4.62 ^[6]^* | | |  | |  | | 5.35 ^[8, 9]^ | |  | |  | | | | | |
| **c** | Ph | | 4-Cl-Ph | | 4.70 ^[3]^ | |  | | |  | | |  | |  | |  | |  | |  | | | | | |
| **d** | 4-Cl-Ph | | 4-Cl-Ph | | 6.39 ^[3]^  6.54 ^[4]^  6.74 ^[3],c^ | | 6.39 ^[3]^  7.00 ^[4]^ | | |  | | |  | |  | |  | |  | |  | | | | | |
| **e** | 4-F-Ph | | 4-F-Ph | | 5.11 ^[3]^ | |  | | |  | | |  | |  | | 6.17 ^[10]^ | |  | |  | | | | | |
| **f** | 4-Me-Ph | | 4-Me-Ph | | 5.24 ^[3]^ | |  | | |  | | |  | |  | |  | |  | |  | | | | | |
| **g** | 4-OCF_3_-Ph | | 4-OCF_3_-Ph | | 6.04 ^[3]^ | |  | | |  | | |  | |  | |  | |  | |  | | | | | |
| ^a^ Values obtained for rat hippocampal slices, unless noted otherwise. ^b^ Values obtained for rat forebrain synaptosomes. ^c^ Value for *(R)*-isomer. ^d^ Cl-966. ^e^ NA = no activity. ^f^ Fumarate adduct. ^g^ Value obtained using cloned hGAT1. ^h^ Gluchowski and co-workers developed various triaryl-substituted nipecotic acid derivatives, which are not included because they are not selective for GAT1, see [13]. Carba-analogues with alkene and alkyne linkers have been developed by Wanner and co-workers, see [14-17] ^i^ Value for racemic mixture. ^j^ Wanner and co-workers developed several more photoswichable GAT inhibitors, see [18]. Carba-analogues with alkene and alkyne linkers have been developed as well, see [19]. | | | | | | | | | | | | | | | | | | | | | | | | | | |
| ***Table S1 (continued):*** *SAR of GAT1 inhibitors with an ether linker* | | | | | | | | | | | | | | | | | | | | | | | | | | |
|  |  | |  | | **1a** | | **1b** | | | **1c** | | | **1d** | | **1e** | | **1f** | | **1g** | | **1h** | | | | | |
|  |  | |  | |  | | | | |  | | |  | |  | |  | | | |  | | | | | |
| **Entry** | **R_1_** | | **R_2_** | | **Nip pIC_50_^a^** | | **Guv**  **pIC_50_ ^a^** | | | **pIC_50_ ^b^** | | | **pIC_50_^b^** | | **pIC_50_ ^b^** | | **Nip**  **pIC_50_ ^b^** | | **Guv**  **pIC_50_^b^** | | **pIC_50_ ^b^** | | | | | |
| **h** | 4-CF_3_-Ph | | 4-CF_3_-Ph | | 6.15 ^[3, 4]^ | | 6.49 ^[3],d^  6.35 ^[4],d^ | | |  | | |  | |  | |  | |  | |  | | | | | |
| **i** | 4-OMe-Ph | | 4-OMe-Ph | | 5.52 ^[1],b^ | |  | | |  | | |  | |  | |  | |  | |  | | | | | |
| **j** | Ph | | 3,4-di-Cl-Ph | | 5.92 ^[3]^ | |  | | |  | | |  | |  | |  | |  | |  | | | | | |
| **k** | 3,4-di-Cl-Ph | | 3,4-di-Cl-Ph | | 5.60 ^[3]^ | |  | | |  | | |  | |  | |  | |  | |  | | | | | |
| **l** | 2-Me-4-Cl-Ph | | 2-Me-4-Cl-Ph | | 5.49 ^[3]^ | |  | | |  | | |  | |  | |  | |  | |  | | | | | |
| **m** | Ph | | 3-CF_3_-4-Cl-Ph | | 6.07 ^[4]^ | |  | | |  | | |  | |  | |  | |  | |  | | | | | |
| **n** | 4-Cl-Ph | | 3-CF_3_-4-Cl-Ph | | 5.85 ^[3]^ | |  | | |  | | |  | |  | |  | |  | |  | | | | | |
| **o** | 3-CF_3_-Ph | | Ph | |  | |  | | |  | | |  | |  | | 7.37 ^[11]^ | |  | |  | | | | | |
| **p** | 2-Me-Ph | | Ph | |  | |  | | |  | | |  | |  | | 7.07 ^[10]^ | |  | |  | | | | | |
| **q** | 2-Me-Ph | | 2-Me-Ph | | 6.41 ^[1],b^ | |  | | |  | | |  | |  | | 6.45 ^[10]^ | |  | |  | | | | | |
| **r** | 2-Me-Ph | | 3-OMe-Ph | |  | |  | | |  | | |  | |  | | 7.16 ^[11]^ | |  | |  | | | | | |
| **s** | Ph | | 3-OMe-Ph | |  | |  | | |  | | |  | |  | | 7.12 ^[11],f^ | |  | |  | | | | | |
| **t** | Cyclohexyl | | 3,4-di-Cl-Ph | | 5.96 ^[3]^ | |  | | |  | | |  | |  | |  | |  | |  | | | | | |
| **u** | Furan-2-yl | | 4-Cl-Ph | | 5.06 ^[3]^ | |  | | |  | | |  | |  | |  | |  | |  | | | | | |
| **v** | Thien-2-yl | | 4-Cl-Ph | | 5.44 ^[3]^ | |  | | |  | | |  | |  | |  | |  | |  | | | | | |
| ^a^ Values obtained for rat hippocampal slices, unless noted otherwise. ^b^ Values obtained for rat forebrain synaptosomes. ^c^ Value for *(R)*-isomer. ^d^ Cl-966. ^e^ NA = no activity. ^f^ Fumarate adduct. ^g^ Value obtained using cloned hGAT1. ^h^ Gluchowski and co-workers developed various triaryl-substituted nipecotic acid derivatives, which are not included because they are not selective for GAT1, see [13]. Carba-analogues with alkene and alkyne linkers have been developed by Wanner and co-workers, see [14-17] ^i^ Value for racemic mixture. ^j^ Wanner and co-workers developed several more photoswichable GAT inhibitors, see [18]. Carba-analogues with alkene and alkyne linkers have been developed as well, see [19]. | | | | | | | | | | | | | | | | | | | | | | | | | | |
| ***Table S1 (continued):*** *SAR of GAT1 inhibitors with an ether linker* | | | | | | | | | | | | | | | | | | | | | | | | | | |
|  |  | |  | | **1a** | | | **1b** | | **1c** | | | **1d** | | **1e** | | **1f** | | **1g** | | **1h** | | | | | |
|  |  | |  | |  | | | | |  | | |  | |  | |  | | | |  | | | | | |
| **Entry** | **R_1_** | | **R_2_** | | **Nip pIC_50_^a^** | | | **Guv**  **pIC_50_ ^a^** | | **pIC_50_ ^b^** | | | **pIC_50_^b^** | | **pIC_50_ ^b^** | | **Nip**  **pIC_50_ ^b^** | | **Guv**  **pIC_50_^b^** | | **pIC_50_ ^b^** | | | | | |
| **w** | Fluorenyl | | Ph | | 5.52 ^[13],g,h^ | | |  | |  | | |  | |  | |  | |  | |  | | | | | |
| **x** | Fluorenyl | | | | NA ^[5],b,e^ | | |  | |  | | |  | |  | |  | |  | |  | | | | | |
| **y** |  | | | |  | | |  | |  | | |  | |  | | 6.25 ^[5]^ | | 5.92 ^[5]^ | |  | | | | | |
| **z** |  | | | | 5.80 ^[3]^ | | |  | |  | | |  | |  | |  | |  | |  | | | | | |
| **aa** |  | | | | 5.60 ^[3]^ | | |  | |  | | |  | |  | |  | |  | |  | | | | | |
| **ab** | Naphthyl | | Ph | |  | | |  | |  | | |  | | 5.10 ^[8]^, 5.19 ^[9]^ | |  | |  | |  | | | | | |
| **ac** | Ph | | CH_2_-Ph | |  | | |  | | 5.16 ^[6, 8]^ | | |  | | 6.49 ^[8, 9]^  *Meta derivative:*  < 5.52 ^[9]^ | |  | |  | | 7.00 ^[8, 9]^ | | | | | |
| **ad** | Ph | | C=CH-Ph | |  | | |  | |  | | | *E:* 6.30 ^[8, 9]^  *Z:* <5.52 ^[8]^, 5.70 ^[9]^ | |  | |  | |  | |  | | | | | |
| **ae** | Ph | | N=N-Ph | |  | | |  | |  | | |  | |  | |  | |  | | 4.97 / 5.26 ^[18],i,j^ | | | | | |
| ^a^ Values obtained for rat hippocampal slices, unless noted otherwise. ^b^ Values obtained for rat forebrain synaptosomes. ^c^ Value for *(R)*-isomer. ^d^ Cl-966. ^e^ NA = no activity. ^f^ Fumarate adduct. ^g^ Value obtained using cloned hGAT1. ^h^ Gluchowski and co-workers developed various triaryl-substituted nipecotic acid derivatives, which are not included because they are not selective for GAT1, see [13]. Carba-analogues with alkene and alkyne linkers have been developed by Wanner and co-workers, see [14-17] ^i^ Value for racemic mixture. ^j^ Wanner and co-workers developed several more photoswichable GAT inhibitors, see [18]. Carba-analogues with alkene and alkyne linkers have been developed as well, see [19]. | | | | | | | | | | | | | | | | | | | | | | | | | | |
| ***Table S2:*** *SAR of GAT1 inhibitors with a vinyl ether linker* | | | | | | | | | | | | | | | | | | | | | | | | | |  |
|  |  |  | | **2a** | | | | | **2b** | | | **2c** | | **2d** | | **2e** | | **2f** | | **2g** | | **2h** | | |  |  |
|  |  |  | |  | | | | |  | | |  | |  | |  | | | |  | | | | |  |  |
| **Entry** | **R_1_** | **R_2_** | | **pK_i_^a^** | | **pIC_50_^a^** | | | **pK_i_^a^** | | **pIC_50_^a^** | **pIC_50_^a^** | | **pIC_50_^a^** | | ***(E)*-pIC_50_^b^** | | ***(Z)*-IC_50_^b^** | | ***(E)*-IC_50_^b^** | | ***(Z)*-IC_50_^b^** | | |  |  |
| **a** | Ph | Ph | | 7.07 ^[20]^ | | 6.98 ^[7]^ | | |  | |  | 6.59 ^[10, 11]^ | |  | | 4.60 ^[21]^ | | 5.15 ^[21]^ | | 5.38 ^[21]^ | | 6.40 ^[21]^ | | |  |  |
| **b** | 2-Me-Ph | Ph | | 7.68 ^[20]^ | |  | | |  | |  | 6.63 ^[10]^ | |  | |  | |  | |  | |  | | |  |  |
| **c** | Ph | 2-Me-Ph | | 7.31 ^[20]^ | |  | | |  | |  |  | |  | |  | |  | |  | |  | | |  |  |
| **d** | 2-Me-Ph | 2-Me-Ph | | 7.72 ^[20]^ | | 7.82 ^[7]^ | | | 7.59 ^[20]^ | | 7.56 ^[7]^ | 6.94 ^[10]^ | | 7.12 ^[10]^, 7.33 ^[11]^ | |  | |  | |  | |  | | |  |  |
| **e** | 3-Me-2-Thi ^c^ | 2-Me-Ph | | 7.77 ^[20]^ | |  | | |  | |  |  | |  | |  | |  | |  | |  | | |  |  |
| **f** | 3-Me-2-Thi ^c^ | 3-Me-2-Thi ^c^ | | 7.85 ^[20]^ | | 8.10 ^[7]^ | | |  | |  |  | |  | |  | |  | |  | |  | | |  |  |
| **g** | 2-Me-Ph | 2-F-Ph | |  | |  | | | 7.72 ^[20]^ | |  |  | |  | |  | |  | |  | |  | | |  |  |
| **h** | 2-Me-Ph | 3-F-Ph | | 7.55 ^[20]^ | |  | | |  | |  |  | |  | |  | |  | |  | |  | | |  |  |
| **i** | 3-F-Ph | 2-Me-Ph | | 7.32 ^[20]^ | |  | | |  | |  |  | |  | |  | |  | |  | |  | | |  |  |
| **j** | 2-Me-Ph | 2-Me-4-F-Ph | | 7.52 ^[20]^ | | 7.52 ^[7]^ | | |  | |  |  | |  | |  | |  | |  | |  | | |  |  |
| **k** | 2-Me-4-F-Ph | 2-Me-4-F-Ph | | 7.21 ^[20]^ | |  | | | 7.10 ^[20]^ | |  |  | |  | |  | |  | |  | |  | | |  |  |
| **l** | 2-Me-Ph | 2-Cl-Ph | | 7.52 ^[20]^ | | 7.52 ^[7]^ | | |  | |  |  | |  | |  | |  | |  | |  | | |  |  |
| **m** | 3-Cl-Ph | 2-Me-Ph | |  | |  | | | 7.19 ^[20]^ | |  |  | |  | |  | |  | |  | |  | | |  |  |
| **n** | 2-Me-Ph | 3-OMe-Ph | | 7.08 ^[20]^ | |  | | |  | |  |  | |  | |  | |  | |  | |  | | |  |  |
| **o** | 2-Et-Ph | 2-Et-Ph | | 7.49 ^[20]^ | |  | | | 7.33 ^[20]^ | | 7.33 ^[7]^ |  | |  | |  | |  | |  | |  | | |  |  |
| **p** | 3-F-Ph | 3-F-Ph | |  | |  | | | 6.46 ^[20]^ | |  |  | |  | |  | |  | |  | |  | | |  |  |
| **q** | 2,5-di-F-Ph | 2,4-di-F-Ph | | 7.29 ^[20]^ | |  | | |  | |  |  | |  | |  | |  | |  | |  | | |  |  |
| **r** | 2,4-di-F-Ph | 2,4-di-F-Ph | | 6.88 ^[20]^ | |  | | |  | |  |  | |  | |  | |  | |  | |  | | |  |  |
| **s** | 2,5-di-F-Ph | 2,5-di-F-Ph | | 6.75 ^[20]^ | |  | | |  | |  |  | |  | |  | |  | |  | |  | | |  |  |
| ^a^ Values obtained for rat forebrain synaptosomes. ^b^ Values are obtained in uptake assays with an assay system based on HEK293 cells stably expressing mGAT1. ^c^ 2-Thi = Thien-2-yl. ^d^ Percentage inhibition at 100 µM. ^e^ SB = Single bond, DB = Double bond. | | | | | | | | | | | | | | | | | | | | | | | | | |  |
| ***Table S2 (continued):*** *SAR of GAT1 inhibitors with a vinyl ether linker* | | | | | | | | | | | | | | | | | | | | | | | | | |  |
|  |  |  | | **2a** | | | | | **2b** | | | **2c** | | **2d** | | **2e** | | **2f** | | **2g** | | **2h** | | | |  |
|  |  |  | |  | | | | |  | | |  | |  | |  | | | |  | | | | |  |  |
| **Entry** | **R_1_** | **R_2_** | | **pK_i_^a^** | | **pIC_50_^a^** | | | **pK_i_^a^** | | **pIC_50_^a^** | **pIC_50_^a^** | | **pIC_50_^a^** | | ***(E)*-pIC_50_^b^** | | ***(Z)*-IC_50_^b^** | | ***(E)*-IC_50_^b^** | | ***(Z)*-IC_50_^b^** | |  |  |  |
| **t** | Ph | 2-Cl-Ph | | 7.92 ^[20]^ | |  | | |  | |  |  | |  | |  | |  | |  | |  | |  |  |  |
| **u** | 2-Cl-Ph | Ph | | 7.28 ^[20]^ | |  | | |  | |  |  | |  | |  | |  | |  | |  | |  |  |  |
| **v** | 2-Cl-Ph | 2-Cl-Ph | | 7.28 ^[20]^ | |  | | | 7.92 ^[20]^ | |  |  | |  | |  | |  | |  | |  | |  |  |  |
| **w** | 2-Cl-4-F-Ph | 2-Cl-4-F-Ph | | 7.18 ^[20]^ | |  | | |  | |  |  | |  | |  | |  | |  | |  | |  |  |  |
| **x** | 3-Cl-Ph | 3-Cl-Ph | | 6.70 ^[20]^ | |  | | |  | |  |  | |  | |  | |  | |  | |  | |  |  |  |
| **y** | 4-Cl-Ph | 4-Cl-Ph | |  | |  | | |  | |  | 6.02 ^[10]^ | |  | |  | |  | |  | |  | |  |  |  |
| **z** | 4-F-Ph | 4-F-Ph | |  | |  | | |  | |  | 5.84 ^[10]^ | |  | | 5.73 ^[21]^ | | 6.47 ^[21]^ | | 5.34 ^[21]^ | | 5.93 ^[21]^ | |  |  |  |
| **aa** | 4-F-Ph | 3-F-Ph | |  | |  | | |  | |  |  | |  | | 67.5% ^[21],d^ | | 6.10 ^[21]^ | |  | |  | |  |  |  |
| **ab** | 4-F-Ph | 2-F-Ph | |  | |  | | |  | |  |  | |  | | 4.97 ^[21]^ | | 6.12 ^[21]^ | | 5.57 ^[21]^ | | 6.20 ^[21]^ | |  |  |  |
| **ac** | 5-F-Ph | 4-F-Ph | |  | |  | | |  | |  |  | |  | | 4.68 ^[21]^ | | 5.12 ^[21]^ | | 5.05 ^[21]^ | | 5.96 ^[21]^ | |  |  |  |
| **ad** | 5-F-Ph | 3-F-Ph | |  | |  | | |  | |  |  | |  | | 4.53 ^[21]^ | | 5.58 ^[21]^ | | 5.17 ^[21]^ | | 6.52 ^[21]^ | |  |  |  |
| **ae** | 5-F-Ph | 2-F-Ph | |  | |  | | |  | |  |  | |  | | 5.09 ^[21]^ | | 5.52 ^[21]^ | | 4.99 ^[21]^ | | 6.52 ^[21]^ | |  |  |  |
| **af ^e^** |  | | |  | | SB:  5.66 ^[5]^ | | |  | | | SB:  6.11 ^[5]^  6.12 ^[11, 22]^ | | SB: 6.05 ^[11, 22]^  6.34 ^[5]^  DB: 5.88 ^[11, 22]^ | |  | |  | |  | |  | | |  |  |
| **ag** |  | | |  | |  | | |  | | | 5.60 ^[5]^ | |  | |  | |  | |  | |  | | |  |  |
| ^a^ Values obtained for rat forebrain synaptosomes. ^b^ Values are obtained in uptake assays with an assay system based on HEK293 cells stably expressing mGAT1. ^c^ 2-Thi = Thien-2-yl. ^d^ Percentage inhibition at 100 µM. ^e^ SB = Single bond, DB = Double bond. | | | | | | | | | | | | | | | | | | | | | | | | | |  |

| ***Table S3:*** *SAR of GAT1 inhibitors with an oxime linker* | | | | | | | | | | | | | | | | | | | |  |
| --- | --- | --- | --- | --- | --- | --- | --- | --- | --- | --- | --- | --- | --- | --- | --- | --- | --- | --- | --- | --- |
|  |  | |  | | **3a** | | | | **3b** | | | | **3c** | **3d** | | **3e** | | **3f** | | |
|  |  | |  | |  | | | |  | | | |  |  | |  | |  | | |
| **Entry** | **R_1_** | | **R_2_** | | **pK_i_^a^** | | **pIC_50_^a^** | | **pK_i_^a^** | | **pIC_50_^a^** | | **pIC_50_^a^** | **pK_i_^b^** | **pIC_50_^c^** | **pK_i_^b^** | **pIC_50_^c^** | **pK_i_^b^** | **pIC_50_^c^** | |
| **a** | Ph | | Ph | | 7.09 ^[20]^ | | 6.86 ^[23],g^  7.09 ^[23]^ | | 7.33 ^[20],d^ | | 7.32 ^[23],d^ | |  |  |  |  |  | 6.19 ^[24, 25]^ | 5.31 ^[24, 25]^ | |
| **b** | Ph | | 2-F-Ph | |  | |  | |  | |  | |  |  |  | 7.95 ^[26]^ | 6.98 ^[26]^ | 6.91 ^[24]^ | 6.28 ^[24]^ | |
| **c** | Ph | | 4-F-Ph | |  | |  | |  | |  | |  |  |  | 7.65 ^[26]^ | 6.73 ^[26]^ | 6.55 ^[24]^ | 5.75 ^[24]^ | |
| **d** | Ph | | 2-Cl-Ph | |  | |  | |  | |  | |  |  |  | 7.91 ^[26]^ | 6.59 ^[26]^ | 7.18 ^[24]^ | 6.32 ^[24]^ | |
| **e** | Ph | | 4-Cl-Ph | |  | |  | |  | |  | |  |  |  |  |  | 6.76 ^[24]^ | 6.01 ^[24]^ | |
| **f** | Ph | | 2-Me-Ph | |  | |  | |  | |  | |  | 7.17 ^[27]^ | 6.72 ^[27]^ |  |  | 6.67 ^[24]^ | 5.81 ^[24]^ | |
| **g** | Ph | | 4-Me-Ph | |  | |  | |  | |  | |  |  |  |  |  | 6.33 ^[24]^ | 5.47 ^[24]^ | |
| **h** | Ph | | 2,4-di-F-Ph | |  | |  | |  | |  | |  | 7.86 ^[28]^  8.23 ^[28],e^ | 7.07 ^[28]^  6.99 ^[28],e^ | 8.35 ^[26]^ | 7.25 ^[26]^ | 7.45 ^[24]^ | 6.89 ^[24]^ | |
| **i** | Ph | | 2,3-di-F-Ph | |  | |  | |  | |  | |  |  |  | 7.65 ^[26]^ | 6.80 ^[26]^ | 6.69 ^[24]^ | 5.82 ^[24]^ | |
| **j** | Ph | | 3,4-di-F-Ph | |  | |  | |  | |  | |  |  |  |  |  | 6.20 ^[24]^ | 5.48 ^[24]^ | |
| **k** | Ph | | 2,6-di-F-Ph | |  | |  | |  | |  | |  |  |  |  |  | 6.88 ^[24]^ | 6.00 ^[24]^ | |
| **l** | Ph | | 2,4,6-tri-F-Ph | |  | |  | |  | |  | |  | 7.38 ^[27]^ | 6.82 ^[27]^ |  |  | 7.51 ^[24]^ | 6.59 ^[24]^ | |
| **m** | Ph | | 2,4-di-Cl-Ph | |  | |  | |  | |  | |  | 7.98 ^[28]^  8.23 ^[28],e^ | 7.41 ^[28]^  7.22 ^[28],e^ | 8.29 ^[26]^ | 8.27 ^[26]^ | 8.09 ^[24]^ | 7.21 ^[24]^ | |
| **n** | Ph | | 2,4-di-Me-Ph | |  | |  | |  | |  | |  |  |  |  |  | 7.14 ^[24]^ | 6.08 ^[24]^ | |
| **o** | Ph | | 2,4-di-CF_3_-Ph | |  | |  | |  | |  | |  |  |  |  |  | 6.77 ^[24]^ | 5.55 ^[24]^ | |
| ^a^ Values obtained for rat forebrain synaptosomes. ^b^ Values are obtained using MS Binding Assays with NNC-711 as MS marker. ^c^ Values are obtained in uptake assays with an assay system based on HEK293 cells stably expressing mGAT1. ^d^ NNC-711. ^e^ Value for *(R)*-isomer. ^f^ 2-Thi = Thien-2-yl ^g^ Value for racemic mixture. ^h^ Value for mixture of E/Z isomers. | | | | | | | | | | | | | | | | | | | | |
| ***Table S3 (continued):*** *SAR of GAT1 inhibitors with an oxime linker* | | | | | | | | | | | | | | | | | | | |  |
|  |  | |  | | **3a** | | | | **3b** | | | | **3c** | **3d** | | **3e** | | **3f** | | |
|  |  | |  | |  | | | |  | | | |  |  | |  | |  | | |
| **Entry** | **R_1_** | | **R_2_** | | **pK_i_^a^** | | **pIC_50_^a^** | | **pK_i_^a^** | | **pIC_50_^a^** | | **pIC_50_^a^** | **pK_i_^b^** | **pIC_50_^c^** | **pK_i_^b^** | **pIC_50_^c^** | **pK_i_^b^** | **pIC_50_^c^** | |
| **p** | Ph | | 2-F-4-Cl-Ph | |  | |  | |  | |  | |  |  |  | 8.05 ^[26]^ | 7.14 ^[26]^ | 7.61 ^[24]^ | 6.90 ^[24]^ | |
| **q** | Ph | | 2-Cl-4-F-Ph | |  | |  | |  | |  | |  | 8.00 ^[28]^  8.55 ^[28],e^ | 7.18 ^[28]^  7.49 ^[28],e^ | 7.87 ^[26]^ | 7.00 ^[26]^ | 7.84 ^[24]^ | 6.54 ^[24]^ | |
| **r** | Ph | | 2-F-6-OMe-Ph | |  | |  | |  | |  | |  |  |  |  |  | 6.35 ^[24]^ | 5.65 ^[24]^ | |
| **s** | Ph | | 2-OMe-4-F-Ph | |  | |  | |  | |  | |  |  |  |  |  | 6.12 ^[24]^ | 5.13 ^[24]^ | |
| **t** | Ph | | CH_2_-Ph | |  | |  | |  | |  | |  |  |  |  |  | 5.54 ^[25]^ | 5.19 ^[25]^ | |
| **u** | Ph | | O-Ph | |  | |  | |  | |  | |  |  |  |  |  | 5.45 ^[25]^ | 4.88 ^[25]^ | |
| **v** | Ph | | 2-Thi ^f^ | |  | |  | |  | |  | |  |  |  |  |  | 6.23 ^[25]^ | 5.54 ^[25]^ | |
| **w** | Ph | | Furan-2-yl | |  | |  | |  | |  | |  |  |  |  |  | 5.58 ^[25]^ | 4.90 ^[25]^ | |
|  | Ph | | 3-Me-1,2,4-Oxadiazol-5-yl | |  | |  | |  | |  | |  |  |  |  |  | 4.48 ^[25]^ | 4.02 ^[25]^ | |
| **x** | 5-F-Ph | | 2-F-Ph | |  | |  | |  | |  | |  |  |  | 7.65 ^[26]^ | 6.77 ^[26]^ | 6.58 ^[24]^ | 5.76 ^[24]^ | |
| **y** | 5-F-Ph | | 2-Cl-Ph | |  | |  | |  | |  | |  |  |  | 7.67 ^[26]^ | 6.37 ^[26]^ | 6.98 ^[24]^ | 5.91 ^[24]^ | |
| **z** | 5-F-Ph | | 4-Cl-Ph | |  | |  | |  | |  | |  |  |  |  |  | 6.63 ^[24]^ | 5.71 ^[24]^ | |
| **aa** | 5-F-Ph | | 2,4-di-Cl-Ph | |  | |  | |  | |  | |  | 7.77 ^[28]^ | 6.77 ^[28]^ | 7.91 ^[26]^ | 6.95 ^[26]^ |  |  | |
| **ab** | 2-Thi ^f^ | | 2,4-di-Cl-Ph | |  | |  | |  | |  | |  | 7.55 ^[28]^ | 7.93 ^[28]^ | 8.25 ^[26]^ | 7.16 ^[26]^ |  |  | |
| **ac** | 2-Me-Ph | | 2-Me-Ph | | 7.15 ^[20]^ | |  | | 7.26 ^[20]^ | |  | |  |  |  |  |  |  |  | |
| **ad** | 2-Me-Ph | | 2-F-Ph | | 7.39 ^[20]^ | |  | |  | |  | |  |  |  |  |  |  |  | |
| **ae** | 2-Me-Ph | | 2-Cl-Ph | | 6.69 ^[20]^ | |  | |  | |  | |  |  |  |  |  |  |  | |
| ^a^ Values obtained for rat forebrain synaptosomes. ^b^ Values are obtained using MS Binding Assays with NNC-711 as MS marker. ^c^ Values are obtained in uptake assays with an assay system based on HEK293 cells stably expressing mGAT1. ^d^ NNC-711. ^e^ Value for *(R)*-isomer. ^f^ 2-Thi = Thien-2-yl ^g^ Value for racemic mixture. ^h^ Value for mixture of E/Z isomers. | | | | | | | | | | | | | | | | | | | | |
| ***Table S3 (continued):*** *SAR of GAT1 inhibitors with an oxime linker* | | | | | | | | | | | | | | | | | | | | |
|  |  | |  | | **3a** | | | | **3b** | | | | **3c** | **3d** | | **3e** | | **3f** | | |
|  |  | |  | |  | | | |  | | | |  |  | |  | |  | | |
| **Entry** | **R_1_** | | **R_2_** | | **pK_i_^a^** | | **pIC_50_^a^** | | **pK_i_^a^** | | **pIC_50_^a^** | | **pIC_50_^a^** | **pK_i_^b^** | **pIC_50_^c^** | **pK_i_^b^** | **pIC_50_^c^** | **pK_i_^b^** | **pIC_50_^c^** | |
| **af** | 2-Me-Ph | | 3-Cl-Ph | |  | |  | | 6.83 ^[20]^ | |  | |  |  |  |  |  |  |  | |
| **ag** | 2-Me-Ph | | 3-Me-2-Thi ^f^ | | 7.06 ^[20]^ | |  | |  | |  | |  |  |  |  |  |  |  | |
| **ah** | 2-Me-Ph | | 4-Me-2-Thi ^f^ | | 6.11 ^[20]^ | |  | |  | |  | |  |  |  |  |  |  |  | |
|  | 2-Me-Ph | | H | |  | | <5.05 ^[29]^ | |  | |  | |  |  |  |  |  |  |  | |
| **ai** | 3-Me-2-Thi ^f^ | | 2-Me-Ph | | 7.15 ^[20]^ | | 7.36 ^[23],h^  7.24 ^[23],h,g^ | |  | |  | |  |  |  |  |  |  |  | |
| **aj** | 3-Me-2-Thi ^f^ | | 2-Et-Ph | |  | | 7.28 ^[23],h^ | |  | |  | |  |  |  |  |  |  |  | |
| **ak** | 3-Me-2-Thi ^f^ | | 2-Thi ^f^ | | 7.13 ^[20]^ | | 7.19 ^[23],h^ | |  | |  | |  |  |  |  |  |  |  | |
| **al** | 3-Me-2-Thi ^f^ | | 3-Me-2-Thi ^f^ | | 7.39 ^[20]^ | | 7.52 ^[23]^ | |  | | 7.23 ^[23]^ | |  |  |  |  |  |  |  | |
| **am** | 3-Me-2-Thi ^f^ | | 2-Me-4-Cl-Ph | | 7.05 ^[20]^ | |  | |  | |  | |  |  |  |  |  |  |  | |
| **an** | 3-Me-2-Thi ^f^ | | 2,4-di-Cl-Ph | | 7.48 ^[20]^ | |  | |  | |  | |  |  |  |  |  |  |  | |
| **ao** | 3-Me-2-Thi ^f^ | | 3-OMe-Ph | | 7.06 ^[20]^ | |  | |  | |  | |  |  |  |  |  |  |  | |
| **ap** | 4-Me-2-Thi ^f^ | | 2-Me-Ph | | 6.58 ^[20]^ | |  | |  | |  | |  |  |  |  |  |  |  | |
| **aq** | 3-Et-2-Thi ^f^ | | 3-Et-2-Thi ^f^ | | 7.06 ^[20]^ | |  | |  | |  | |  |  |  |  |  |  |  | |
| **ar** | 3-Et-Ph | | 3-Et-Ph | |  | |  | | 6.74 ^[20]^ | |  | |  |  |  |  |  |  |  | |
| **as** | 2-F-Ph | | 3-F-Ph | | 6.67 ^[20]^ | |  | |  | |  | |  |  |  |  |  |  |  | |
| **at** | 2,4-di-F-Ph | | 2,4-di-F-Ph | | 6.99 ^[20]^ | |  | |  | |  | |  |  |  |  |  |  |  | |
| **au** | 2,4-di-F-Ph | | 2,5-di-F-Ph | | 7.28 ^[20]^ | |  | |  | |  | |  |  |  |  |  |  |  | |
| ^a^ Values obtained for rat forebrain synaptosomes. ^b^ Values are obtained using MS Binding Assays with NNC-711 as MS marker. ^c^ Values are obtained in uptake assays with an assay system based on HEK293 cells stably expressing mGAT1. ^d^ NNC-711. ^e^ Value for *(R)*-isomer. ^f^ 2-Thi = Thien-2-yl ^g^ Value for racemic mixture. ^h^ Value for mixture of E/Z isomers. | | | | | | | | | | | | | | | | | | | | |
| ***Table S3 (continued):*** *SAR of GAT1 inhibitors with an oxime linker* | | | | | | | | | | | | | | | | | | | | |
|  |  | | |  | **3a** | | | | **3b** | | | | **3c** | **3d** | | **3e** | | **3f** | | |
|  |  | | |  |  | | | |  | | | |  |  | |  | |  | | |
| **Entry** | **R_1_** | | | **R_2_** | **pK_i_^a^** | **pIC_50_^a^** | | | **pK_i_^a^** | **pIC_50_^a^** | | | **pIC_50_^a^** | **pK_i_^b^** | **pIC_50_^c^** | **pK_i_^b^** | **pIC_50_^c^** | **pK_i_^b^** | **pIC_50_^c^** | |
| **av** | 2-Cl-Ph | | | 2-Cl-Ph | 7.41 ^[20]^ |  | | |  |  | | |  |  |  |  |  |  |  | |
| **aw** | 3-Cl-Ph | | | 3-Cl-Ph | 6.52 ^[20]^ |  | | |  |  | | |  |  |  |  |  |  |  | |
| **ax** | 2-Cl-4-F-Ph | | | 2-Cl-4-F-Ph | 7.00 ^[20]^ |  | | |  |  | | |  |  |  |  |  |  |  | |
| **ay** | 1-Me-Pyrrol-2-yl | | | Ph | 7.23 ^[20]^ |  | | |  |  | | |  |  |  |  |  |  |  | |
| **az** | Pyridin-2-yl | | | Ph | 5.68 ^[20],g^ |  | | |  |  | | |  |  |  |  |  |  |  | |
| **ba** | Cyclopropyl | | | Ph |  | 5.91 ^[8],h^  6.02 ^[29],h^ | | |  |  | | |  |  |  |  |  |  |  | |
| **bb** | Isobutyl | | | Ph |  | 6.55 ^[8, 29],h^ | | |  |  | | |  |  |  |  |  |  |  | |
| **bc** | CH_2_-Ph | | | Ph |  | 6.87 ^[8, 29],h^ | | |  |  | | |  |  |  |  |  |  |  | |
| **bd** | CH_2_-4-F-Ph | | | 4-F-Ph |  | 6.03 ^[8, 29],h^ | | |  |  | | |  |  |  |  |  |  |  | |
| **be** | CH_2_-2-F-Ph | | | 4-F-Ph |  | 7.10 ^[8, 29],h^ | | |  |  | | |  |  |  |  |  |  |  | |
| **bf** | CH_2_-Ph | | | CH_2_-Ph |  | 5.99 ^[8, 29]^ | | |  |  | | |  |  |  |  |  |  |  | |
| **bg** | Cyclohexyl | | | Ph |  |  | | |  |  | | | 6.49 ^[8, 30]^ |  |  |  |  |  |  | |
| **bh** | Cyclohexyl | | | C=C-Ph |  |  | | |  |  | | | 5.01 ^[8, 30]^ |  |  |  |  |  |  | |
| **bi** | Cyclohex-2-enyl | | | Ph |  |  | | |  |  | | | 6.46 ^[8]^  6.38 ^[30]^ |  |  |  |  |  |  | |
| ^a^ Values obtained for rat forebrain synaptosomes. ^b^ Values are obtained using MS Binding Assays with NNC-711 as MS marker. ^c^ Values are obtained in uptake assays with an assay system based on HEK293 cells stably expressing mGAT1. ^d^ NNC-711. ^e^ Value for *(R)*-isomer. ^f^ 2-Thi = Thien-2-yl ^g^ Value for racemic mixture. ^h^ Value for mixture of E/Z isomers. | | | | | | | | | | | | | | | | | | | | |
| ***Table S3 (continued):*** *SAR of GAT1 inhibitors with an oxime linker* | | | | | | | | | | | | | | | | | | | | |
|  |  | | |  | **3a** | | | **3b** | | | | **3c** | | **3d** | | **3e** | | **3f** | | |
|  |  | | |  |  | | |  | | | |  | |  | |  | |  | | |
| **Entry** | **R_1_** | | | **R_2_** | **pK_i_^a^** | **pIC_50_^a^** | | **pK_i_^a^** | | **pIC_50_^a^** | | **pIC_50_^a^** | | **pK_i_^b^** | **pIC_50_^c^** | **pK_i_^b^** | **pIC_50_^c^** | **pK_i_^b^** | **pIC_50_^c^** | |
| **bj** | Ph | | | Cyclopenten-1-yl |  | 6.71 ^[29]^ | |  | |  | |  | |  |  |  |  |  |  | |
| **bk** | Ph | | | CF_3_ |  | 5.35 ^[29]^ | |  | |  | |  | |  |  |  |  |  |  | |
| **bl** | Ph | | | (1-methoxy)  methyl |  | 5.70 ^[29]^ | |  | |  | |  | |  |  |  |  |  |  | |
| **bm** | Ph | | | (4-methoxy)  butyl |  | <5.52 ^[29],h^ | |  | |  | |  | |  |  |  |  |  |  | |
| **bn** | Ph | | | Heptyl |  | 5.96 ^[29]^ | |  | |  | |  | |  |  |  |  |  |  | |
| **bo** | Heptyl | | | Ph |  | 5.66 ^[29]^ | |  | |  | |  | |  |  |  |  |  |  | |
| **bp** |  | | | Ph |  |  | |  | |  | | 6.06 ^[8, 30]^ | |  |  |  |  |  |  | |
| **bq** |  | | | Ph |  |  | |  | |  | | 5.85 ^[8, 30]^ | |  |  |  |  |  |  | |
| **br** |  | | | |  | 5.95 ^[22]^ | | 5.94 ^[5]^ | |  | |  | |  |  |  |  |  |  | |
| ^a^ Values obtained for rat forebrain synaptosomes. ^b^ Values are obtained using MS Binding Assays with NNC-711 as MS marker. ^c^ Values are obtained in uptake assays with an assay system based on HEK293 cells stably expressing mGAT1. ^d^ NNC-711. ^e^ Value for *(R)*-isomer. ^f^ 2-Thi = Thien-2-yl ^g^ Value for racemic mixture. ^h^ Value for mixture of E/Z isomers. | | | | | | | | | | | | | | | | | | | | |
| ***Table S3 (continued):*** *SAR of GAT1 inhibitors with an oxime linker* | | | | | | | | | | | | | | | | | | | | |
|  |  |  | | | **3a** | | | **3b** | | | | **3c** | | **3d** | | **3e** | | **3f** | | |
|  |  |  | | |  | | |  | | | |  | |  | |  | |  | | |
| **Entry** | **R_1_** | **R_2_** | | | **pK_i_^a^** | **pIC_50_^a^** | | **pK_i_^a^** | | **pIC_50_^a^** | | **pIC_50_^a^** | | **pK_i_^b^** | **pIC_50_^c^** | **pK_i_^b^** | **pIC_50_^c^** | **pK_i_^b^** | **pIC_50_^c^** | |
| **bs** |  | | | |  |  | | 5.26 ^[5]^ | |  | |  | |  |  |  |  |  |  | |
| **bt** |  | | | |  | 6.79 ^[22]^ | |  | |  | |  | |  |  |  |  |  |  | |
| **bu** |  | | | |  | 6.12 ^[8],h^  6.10 ^[29],h^ | |  | |  | | ` | |  |  |  |  |  |  | |
| **bv** |  | | | |  | 6.54 ^[8, 29],h^ | |  | |  | |  | |  |  |  |  |  |  | |
| ^a^ Values obtained for rat forebrain synaptosomes. ^b^ Values are obtained using MS Binding Assays with NNC-711 as MS marker. ^c^ Values are obtained in uptake assays with an assay system based on HEK293 cells stably expressing mGAT1. ^d^ NNC-711. ^e^ Value for *(R)*-isomer. ^f^ 2-Thi = Thien-2-yl ^g^ Value for racemic mixture. ^h^ Value for mixture of E/Z isomers. | | | | | | | | | | | | | | | | | | | | |

| ***Table S3 (continued):*** *SAR of GAT1 inhibitors with an oxime linker* | | | | | | | | | | | | | |
| --- | --- | --- | --- | --- | --- | --- | --- | --- | --- | --- | --- | --- | --- |
|  |  |  | **3a** | | **3b** | | **3c** | **3d** | | **3e** | | **3f** | |
|  |  |  |  | |  | |  |  | |  | |  | |
| **Entry** | **R_1_** | **R_2_** | **pK_i_^a^** | **pIC_50_^a^** | **pK_i_^a^** | **pIC_50_^a^** | **pIC_50_^a^** | **pK_i_^b^** | **pIC_50_^c^** | **pK_i_^b^** | **pIC_50_^c^** | **pK_i_^b^** | **pIC_50_^c^** |
| **bw** |  | |  | 6.57 ^[8],h^  6.63 ^[29],h^ |  |  |  |  |  |  |  |  |  |
| **bx** |  | |  | 6.34 ^[8],h^  6.47 ^[29],h^ |  |  |  |  |  |  |  |  |  |
| **by** |  | |  | 6.66 ^[23],h^ |  |  |  |  |  |  |  |  |  |
| ^a^ Values obtained for rat forebrain synaptosomes. ^b^ Values are obtained using MS Binding Assays with NNC-711 as MS marker. ^c^ Values are obtained in uptake assays with an assay system based on HEK293 cells stably expressing mGAT1. ^d^ NNC-711. ^e^ Value for *(R)*-isomer. ^f^ 2-Thi = Thien-2-yl ^g^ Value for racemic mixture. ^h^ Value for mixture of E/Z isomers. | | | | | | | | | | | | | |

| ***Table S4:*** *SAR of GAT1 inhibitors with an amine linker* | | | | | | | | |
| --- | --- | --- | --- | --- | --- | --- | --- | --- |
|  |  |  |  | **4a** | **4b** | **4c** | **4d** | **4e** |
|  |  |  |  |  |  |  |  |  |
| **Entry** | **n** | **R_1_** | **R_2_** | **pIC_50_^a^** | **pIC_50_^a^** | **pIC_50_^a^** | **pIC_50_^a^** | **pIC_50_^a^** |
| **a** | 2 | Ph | Ph |  |  | 7.05 ^[10]^, 6.97 ^[11]^ |  | <5.05 ^[10]^ |
| **b** | 3 | Ph | Ph | <5.05 ^[10]^ |  |  | <5.52 ^[10]^ |  |
| **c** | 4 | Ph | Ph | 5.58 ^[10]^ |  | 6.52 ^[10]^ |  |  |
| **d** | 5 | Ph | Ph | 5.86 ^[10]^ |  |  |  |  |
| **e** | 6 | Ph | Ph | 6.18 ^[10, 11]^ |  |  |  |  |
| **f** | 7 | Ph | Ph | 6.64 ^[10, 11]^ | <5.05 ^[10]^ |  |  |  |
| **g** | 8 | Ph | Ph | 5.89 ^[10]^ |  |  |  |  |
| **h** | 2 | *i*-Butyl^b^ | Ph |  |  | 6.55 ^[8, 9]^ |  |  |
| **i** | 2 | *i*-Butyl^b^ | 2-Cl-4-F-Ph |  |  | 6.83 ^[8, 9]^ |  |  |
| **j** | 2 | Fluorenyl | |  |  | <4.40 ^[5]^ |  |  |
| **k** | 2 |  | |  |  | 4.84 ^[5]^ |  |  |
| **l** | 2 |  | |  |  | 6.51 ^[5, 22]^ |  |  |
| ^a^ Values obtained for rat forebrain synaptosomes. ^b^ *i*-Butyl = isobutyl. ^c^ Value for the *(S)­*-isomer. ^d/e^ Value for ^d^guvacine or ^e^homo-b-proline derivative. ^f^ Wanner and co-workers developed various tricyclic-substituted derivatives, which are not included due to their moderate potencies, see [31]. | | | | | | | | |

| ***Table S4 (Continued):*** *SAR of GAT1 inhibitors with an amine linker* | | | | | | | | |
| --- | --- | --- | --- | --- | --- | --- | --- | --- |
|  |  |  |  | **4a** | **4b** | **4c** | **4d** | **4e** |
|  |  |  |  |  |  |  |  |  |
| **Entry** | **n** | **R_1_** | **R_2_** | **pIC_50_^a^** | **pIC_50_^a^** | **pIC_50_^a^** | **pIC_50_^a^** | **pIC_50_^a^** |
| **m** | 2 |  | |  |  | 6.98 ^[5],c^ |  |  |
|  | 2 |  | |  |  | 6.92 ^[22]^ |  |  |
| **n** | 2 |  | |  |  | 6.74 ^[5]^, 6.65 ^[22]^  6.19 ^[5],d^  7.29 ^[5],e^ |  |  |
| **o** | 3 |  |  |  |  | 6.16 ^[5]^  6.21 ^[22]^ |  |  |
| **p** | 2 |  | |  |  | 6.19 ^[5]^ |  |  |
| **q** | 2 |  | |  |  | 6.07 ^[5]^  6.14 ^[22]^ |  |  |
| ^a^ Values obtained for rat forebrain synaptosomes. ^b^ *i*-Butyl = isobutyl. ^c^ Value for the *(S)­*-isomer. ^d/e^ Value for ^d^guvacine or ^e^homo-b-proline derivative. ^f^ Wanner and co-workers developed various tricyclic-substituted derivatives, which are not included due to their moderate potencies, see [31]. | | | | | | | | |

| ***Table S4 (Continued):*** *SAR of GAT1 inhibitors with an amine linker* | | | | | | | | | | | | | | | | | | | |
| --- | --- | --- | --- | --- | --- | --- | --- | --- | --- | --- | --- | --- | --- | --- | --- | --- | --- | --- | --- |
|  |  |  | |  | **4a** | | | **4b** | | | **4c** | | | **4d** | | | **4e** | | |
|  |  |  | |  |  | | |  | | |  | | |  | | |  | | |
| **Entry** | **n** | **R_1_** | | **R_2_** | **pIC_50_^a^** | | | **pIC_50_^a^** | | | **pIC_50_^a^** | | | **pIC_50_^a^** | | | **pIC_50_^a^** | | |
| **r** | 2 |  | | |  | | |  | | | 7.13 ^[5]^ | | |  | | |  | | |
| **s** | 2 |  | | |  | | |  | | | 7.17 ^[5, 22]^ | | |  | | |  | | |
| **t** | 2 |  | | |  | | |  | | | 6.58 ^[5]^ | | |  | | |  | | |
| **u** | 2 |  | | |  | | |  | | | 6.61 ^[5]^ | | |  | | |  | | |
| **v** | 2 |  | | |  | | |  | | | 5.99 ^[5]^ | | |  | | |  | | |
| **w** | 4 |  | | | 4.40 ^[31],f^ | | |  | | |  | | |  | | |  | | |
| ^a^ Values obtained for rat forebrain synaptosomes. ^b^ *i*-Butyl = isobutyl. ^c^ Value for the *(S)­*-isomer. ^d/e^ Value for ^d^guvacine or ^e^homo-b-proline derivative. ^f^ Wanner and co-workers developed various tricyclic-substituted derivatives, which are not included due to their moderate potencies, see [31]. | | | | | | | | | | | | | | | | | | | |
| ***Table S5:*** *SAR of GAT1 inhibitors with an alkene linker* | | | | | | | | | | | | | | | | | | | |
|  |  | |  | | | **5a** | **5b** | | | **5c** | | | | | **5d** | | | **5e** | |
|  |  | |  | | |  |  | | |  | | | | |  | | |  | |
| **Entry** | **R_1_** | | **R_2_** | | | **pIC_50_^a^** | **pIC_50_ ^a^** | | **pK_i_^b^** | **pIC_50_ ^a^** | | **pK_i_^c^** | | | **pIC_50_ ^a^** | **pK_i_^d^** | | **pIC_50_ ^a^** | **pK_i_^d^** |
| **a** | H | | H | | |  | 3.72 ^[32],e,f^ | | 3.53 ^[32],f^ |  | |  | | | 3.10 ^[32],e,f^ | 2.84 ^[32],f^ | |  |  |
| **b** | Ph | | Ph | | | 5% at 10 μM ^[33],g^ | 6.70 ^[33-35],i^  6.48 ^[1],i^  5.00 ^[3],h,i^  27% at  10 μM ^[33],f,g^ | | 5.88  ^[36],i^ | 5.49 ^[32],e^  6.96 ^[33]^ | | 6.33 ^[32],d^  7.14 ^[37]^ | | | 5.22 ^[32],e,j^  6.42 ^[38],j^  6.47 ^[1],j^  6.68 ^[33-35],j^ | 6.32 ^[32],j^  5.31 ^[36],b,j^ | | 31% at 1 μM ^[33],g^ |  |
| **c** | 2-Thi ^k^ | | Ph | | |  | 6.78 ^[34, 35],l^  6.33 ^[39],l^ | |  | 6.63 ^[39],l^ | |  | | | 6.58 ^[1]^  6.66 ^[39],l^ |  | |  |  |
| **d** | 2-Thi ^k^ | | 2-Thi ^k^ | | |  | 6.59 ^[1]^ | |  | 6.40 ^[40],e^ | |  | | |  |  | |  |  |
| **e** | Ph | | Cyclohexyl | | |  | 6.75 ^[34]^ | |  |  | |  | | |  |  | |  |  |
| **f** | Ph | | 2-F-Ph | | |  |  | |  |  | | 6.43 ^[37]^ | | |  |  | |  |  |
| **g** | Ph | | 4-F-Ph | | |  | 6.55 ^[34, 35],l^ | |  |  | |  | | |  |  | |  |  |
| **h** | Ph | | 2-Cl-Ph | | |  |  | |  |  | | 6.78 ^[37]^ | | |  |  | |  |  |
| **i** | Ph | | 3-Cl-Ph | | |  |  | |  |  | |  | | | 6.55 ^[34, 35],l^ |  | |  |  |
| **j** | Ph | | 4-Cl-Ph | | |  |  | |  |  | |  | | | 6.45 ^[34, 35],l^ |  | |  |  |
| **k** | Ph | | 2-Br-Ph | | |  |  | |  |  | | 6.70 ^[37]^ | | |  |  | |  |  |
| **l** | Ph | | 2-CF_3_-Ph | | |  |  | |  |  | | 5.72 ^[37]^ | | |  |  | |  |  |
| ^a^ Values are obtained for rat forebrain synaptosomes, unless noted otherwise. ^b^ Values obtained for neurons cultured from cerebral cortices of mouse embryos. ^c^ Values are obtained using [^3^H]-Tiagabine radioligand Binding Assays based on HEK293 cells stably expressing hGAT1, unless noted otherwise. ^d^ Values are obtained using MS Binding Assays with NNC-711 as MS marker. ^e^ Values are obtained in uptake assays with mGAT1. ^f^ Value given for the reduced structure (i.e. with butynyl tail). ^g^ Percentage inhibition at given concentration. ^h^ Value is obtained for rat hippocampal slices. ^i^ SKF-89976A. ^j^ SKF-100330A. ^k^ 2-Thi = Thien-2-yl. ^l^ Value for mixed isomers (*E/Z*). ^m^ 2-Pyr = Pyrrol-2-yl. ^n^ Tiagabine. | | | | | | | | | | | | | | | | | | | |
| ***Table S5 (continued):*** *SAR of GAT1 inhibitors with an alkene linker* | | | | | | | | | | | | | | | | | | | |
|  |  | |  | | | **5a** | **5b** | | | **5c** | | | | | **5d** | | | **5e** | |
|  |  | |  | | |  |  | | |  | | | | |  | | |  | |
| **Entry** | **R_1_** | | **R_2_** | | | **pIC_50_^a^** | **pIC_50_ ^a^** | | **pK_i_^b^** | **pIC_50_ ^a^** | | **pK_i_^c^** | | | **pIC_50_ ^a^** | **pK_i_^d^** | | **pIC_50_ ^a^** | **pK_i_^d^** |
| **m** | Ph | | 2-Me-Ph | | |  |  | |  |  | | 7.03 ^[37]^ | | |  |  | |  |  |
| **n** | Ph | | 2-Et-Ph | | |  |  | |  |  | | 6.99 ^[37]^ | | |  |  | |  |  |
| **o** | Ph | | 2-Ph-Ph | | |  |  | |  |  | | 6.17 ^[37]^ | | |  |  | |  |  |
| **p** | Ph | | 2-OMe-Ph | | |  |  | |  |  | | 6.43 ^[37]^ | | |  |  | |  |  |
| **q** | Ph | | 2-OCF_3_-Ph | | |  |  | |  |  | | 6.19 ^[37]^ | | |  |  | |  |  |
| **r** | Ph | | 2,4-di-F-Ph | | |  |  | |  |  | | 6.16 ^[37]^ | | |  |  | |  |  |
| **s** | Ph | | 2-Me-4-F-Ph | | |  |  | |  |  | | 6.42 ^[37]^ | | |  |  | |  |  |
| **t** | Ph | | 1-Et-2-Pyr^m^ | | |  |  | |  |  | |  | | | 6.79 ^[39],l^ |  | |  |  |
| **u** | 2-F-Ph | | Ph | | |  |  | |  |  | | 7.16 ^[37]^ | | |  |  | |  |  |
| **v** | 2-Cl-Ph | | Ph | | |  |  | |  |  | | 7.39 ^[37]^ | | |  |  | |  |  |
| **w** | 2-Br-Ph | | Ph | | |  |  | |  |  | | 7.54 ^[37]^ | | |  |  | |  |  |
| **x** | 2-CF_3_-Ph | | Ph | | |  |  | |  |  | | 7.03 ^[37]^ | | |  |  | |  |  |
| **y** | 2-Me-Ph | | Ph | | |  |  | |  |  | | 7.69 ^[37]^ | | |  |  | |  |  |
| **z** | 3-Me-Ph | | Ph | | |  | 6.43 ^[35],l^ | |  |  | |  | | |  |  | |  |  |
| **aa** | 4-Me-Ph | | Ph | | |  | 6.28 ^[35],l^ | |  |  | |  | | |  |  | |  |  |
| **ab** | 3-Me-2-Thi ^k^ | | Ph | | |  | 6.54 ^[1]^ | |  |  | |  | | |  |  | |  |  |
| **ac** | 3-Me-2-Thi ^k^ | | 2-Thi ^k^ | | |  | 6.96 ^[38],l^ | |  | 7.21 ^[1]^ | |  | | | 6.95 ^[1]^ |  | |  |  |
| ^a^ Values are obtained for rat forebrain synaptosomes, unless noted otherwise. ^b^ Values obtained for neurons cultured from cerebral cortices of mouse embryos. ^c^ Values are obtained using [^3^H]-Tiagabine radioligand Binding Assays based on HEK293 cells stably expressing hGAT1, unless noted otherwise. ^d^ Values are obtained using MS Binding Assays with NNC-711 as MS marker. ^e^ Values are obtained in uptake assays with mGAT1. ^f^ Value given for the reduced structure (i.e. with butynyl tail). ^g^ Percentage inhibition at given concentration. ^h^ Value is obtained for rat hippocampal slices. ^i^ SKF-89976A. ^j^ SKF-100330A. ^k^ 2-Thi = Thien-2-yl. ^l^ Value for mixed isomers (*E/Z*). ^m^ 2-Pyr = Pyrrol-2-yl. ^n^ Tiagabine. | | | | | | | | | | | | | | | | | | | |
| ***Table S5 (continued):*** *SAR of GAT1 inhibitors with an alkene linker* | | | | | | | | | | | | | | | | | | | |
|  |  | |  | | | **5a** | **5b** | | | **5c** | | | | | **5d** | | | **5e** | |
|  |  | |  | | |  |  | | |  | | | | |  | | |  | |
| **Entry** | **R_1_** | | **R_2_** | | | **pIC_50_^a^** | **pIC_50_ ^a^** | | **pK_i_^b^** | **pIC_50_ ^a^** | | **pK_i_^c^** | | | **pIC_50_ ^a^** | **pK_i_^d^** | | **pIC_50_ ^a^** | **pK_i_^d^** |
| **ad** | 2-Et-Ph | | Ph | | |  |  | |  |  | | 7.53 ^[37]^ | | |  |  | |  |  |
| **ae** | 2-Ph-Ph | | Ph | | |  |  | |  |  | | 5.97 ^[37]^ | | |  |  | |  |  |
| **af** | 2-OMe-Ph | | Ph | | |  |  | |  |  | | 7.25 ^[37]^ | | |  |  | |  |  |
| **ag** | 3-OMe-Ph | | Ph | | |  | 6.80 ^[35],l^ | |  |  | |  | | |  |  | |  |  |
| **ah** | 4-OMe-Ph | | Ph | | |  | 5.90 ^[35],l^ | | 4.94 ^[36],l^ |  | |  | | |  |  | |  |  |
| **ai** | 2-OCF_3_-Ph | | Ph | | |  |  | |  |  | | 7.23 ^[37]^ | | |  |  | |  |  |
| **aj** | 2,6-di-F-Ph | | Ph | | |  |  | |  |  | | 6.48 ^[37]^ | | |  |  | |  |  |
| **ak** | 2-Me-4-F-Ph | | Ph | | |  |  | |  |  | | 7.47 ^[37]^ | | |  |  | |  |  |
| **al** | 2,6-di-Me-Ph | | Ph | | |  |  | |  |  | | 7.11 ^[37]^ | | |  |  | |  |  |
| **am** | 1-Me-2-Pyr^m^ | | Ph | | |  |  | |  | 7.17 ^[39],l^ | |  | | | 6.89 ^[1]^  6.90 ^[39],l^ |  | |  |  |
|  | 1-Me-2-Pyr^m^ | | 1-Me-2-Pyr^m^ | | |  | 7.22 ^[38]^ | |  |  | |  | | |  |  | |  |  |
| **an** | 2-F-Ph | | 2-Me-Ph | | |  |  | |  |  | | 7.14 ^[37]^ | | |  |  | |  |  |
| **ao** | 2-F-Ph | | 2-Et-Ph | | |  |  | |  |  | | 7.05 ^[37]^ | | |  |  | |  |  |
| **ap** | 2-F-Ph | | 2-F-Ph | | |  |  | |  |  | | 7.33 ^[37]^ | | |  |  | |  |  |
| **aq** | 2-F-Ph | | 2-Cl-Ph | | |  |  | |  |  | | 6.80 ^[37]^ | | |  |  | |  |  |
| ^a^ Values are obtained for rat forebrain synaptosomes, unless noted otherwise. ^b^ Values obtained for neurons cultured from cerebral cortices of mouse embryos. ^c^ Values are obtained using [^3^H]-Tiagabine radioligand Binding Assays based on HEK293 cells stably expressing hGAT1, unless noted otherwise. ^d^ Values are obtained using MS Binding Assays with NNC-711 as MS marker. ^e^ Values are obtained in uptake assays with mGAT1. ^f^ Value given for the reduced structure (i.e. with butynyl tail). ^g^ Percentage inhibition at given concentration. ^h^ Value is obtained for rat hippocampal slices. ^i^ SKF-89976A. ^j^ SKF-100330A. ^k^ 2-Thi = Thien-2-yl. ^l^ Value for mixed isomers (*E/Z*). ^m^ 2-Pyr = Pyrrol-2-yl. ^n^ Tiagabine. | | | | | | | | | | | | | | | | | | | |
| ***Table S5 (continued):*** *SAR of GAT1 inhibitors with an alkene linker* | | | | | | | | | | | | | | | | | | | |
|  |  | |  | | | **5a** | **5b** | | | **5c** | | | | | **5d** | | | **5e** | |
|  |  | |  | | |  |  | | |  | | | | |  | | |  | |
| **Entry** | **R_1_** | | **R_2_** | | | **pIC_50_^a^** | **pIC_50_ ^a^** | | **pK_i_^b^** | **pIC_50_ ^a^** | | **pK_i_^c^** | | | **pIC_50_ ^a^** | **pK_i_^d^** | | **pIC_50_ ^a^** | **pK_i_^d^** |
| **ar** | 2-F-Ph | | 2-CF_3_-Ph | | |  |  | |  |  | | 6.45 ^[37]^ | | |  |  | |  |  |
| **as** | 2-Me-Ph | | 2-Me-Ph | | |  |  | |  | 7.09 ^[1]^ | | 7.38 ^[37]^ | | |  |  | |  |  |
| **at** | 2-Me-Ph | | 2-F-Ph | | |  |  | |  |  | | 7.83 ^[37]^ | | |  |  | |  |  |
| **au** | 2-Me-Ph | | 2-Cl-Ph | | |  |  | |  |  | | 7.21 ^[37]^ | | |  |  | |  |  |
| **av** | 2-Me-Ph | | 2-CF_3_-Ph | | |  |  | |  |  | | 6.42 ^[37]^ | | |  |  | |  |  |
| **aw** | 2-Me-Ph | | 2-OMe-Ph | | |  |  | |  |  | | 7.15 ^[37]^ | | |  |  | |  |  |
| **ax** | 2-Me-Ph | | 1-Et-2-Pyr^m^ | | |  |  | |  | 7.31 ^[39],l^ | |  | | |  |  | |  |  |
| **ay** | 2-Me-Ph | | 1-Pr-2-Pyr^m^ | | |  |  | |  | 7.07 ^[39],l^ | |  | | |  |  | |  |  |
| **az** | 3-Me-2-Thi ^k^ | | 2-Me-Ph | | |  |  | |  | 7.11 ^[1]^  7.13 ^[39],l^ | |  | | | 6.89 ^[1]^  6.93 ^[39],l^ |  | |  |  |
| **ba** | 3-Me-2-Thi ^k^ | | 3-Me-2-Thi ^k^ | | |  | 7.06 ^[1, 38]^ | |  | 6.36 ^[40],e,n^  6.88 ^[32],e,n^  7.17 ^[1],n^ | | 7.37 ^[32],d,n^  7.77 ^[37],n^ | | | 6.61 ^[32],d^  6.86 ^[1]^ | 7.04 ^[32]^ | |  |  |
| **bb** | 3-Me-2-Thi ^k^ | | 3-OMe-Ph | | |  |  | |  | 7.25 ^[39]31,l^ | |  | | |  |  | |  |  |
| **bc** | 3-Me-2-Thi ^k^ | | 4-OMe-Ph | | |  |  | |  | 5.95 ^[40],e^ | |  | | |  |  | |  |  |
| **bd** | 3-Me-2-Thi ^k^ | | 2-F-Ph | | |  |  | |  | 6.66 ^[39],l^ | |  | | |  |  | |  |  |
| **be** | 3-Me-2-Thi ^k^ | | 3-Cl-Ph | | |  |  | |  | 6.91 ^[39],l^ | |  | | |  |  | |  |  |
| ^a^ Values are obtained for rat forebrain synaptosomes, unless noted otherwise. ^b^ Values obtained for neurons cultured from cerebral cortices of mouse embryos. ^c^ Values are obtained using [^3^H]-Tiagabine radioligand Binding Assays based on HEK293 cells stably expressing hGAT1, unless noted otherwise. ^d^ Values are obtained using MS Binding Assays with NNC-711 as MS marker. ^e^ Values are obtained in uptake assays with mGAT1. ^f^ Value given for the reduced structure (i.e. with butynyl tail). ^g^ Percentage inhibition at given concentration. ^h^ Value is obtained for rat hippocampal slices. ^i^ SKF-89976A. ^j^ SKF-100330A. ^k^ 2-Thi = Thien-2-yl. ^l^ Value for mixed isomers (*E/Z*). ^m^ 2-Pyr = Pyrrol-2-yl. ^n^ Tiagabine. | | | | | | | | | | | | | | | | | | | |
| ***Table S5 (continued):*** *SAR of GAT1 inhibitors with an alkene linker* | | | | | | | | | | | | | | | | | | | |
|  |  | |  | | | **5a** | **5b** | | | **5c** | | | | | **5d** | | | **5e** | |
|  |  | |  | | |  |  | | |  | | | | |  | | |  | |
| **Entry** | **R_1_** | | **R_2_** | | | **pIC_50_^a^** | **pIC_50_ ^a^** | | **pK_i_^b^** | **pIC_50_ ^a^** | | | **pK_i_^c^** | | **pIC_50_ ^a^** | **pK_i_^d^** | | **pIC_50_ ^a^** | **pK_i_^d^** |
| **bf** | 3-Me-2-Thi ^k^ | | 4-Cl-Ph | | |  |  | |  |  | | |  | | 6.07 ^[1]^ |  | |  |  |
| **bg** | 3-Me-2-Thi ^k^ | | 3-Et-Ph | | |  |  | |  | 7.14 ^[39],l^ | | |  | |  |  | |  |  |
| **bh** | 3-Me-2-Thi ^k^ | | 2,4-di-Cl-Ph | | |  |  | |  |  | | |  | | 6.49 ^[1]^ |  | |  |  |
| **bi** | 3-Me-2-Thi ^k^ | | 3,5-di-Cl-Ph | | |  |  | |  |  | | |  | | 5.73 ^[1]^ |  | |  |  |
| **bj** | 3-Me-2-Thi ^k^ | | 2-Me-3-Cl-Ph | | |  |  | |  | 6.98 ^[39],l^ | | |  | |  |  | |  |  |
| **bk** | 3-Me-2-Thi ^k^ | | 2-Me-4-Cl-Ph | | |  |  | |  | 6.95 ^[1]^  7.05 ^[39],l^ | | |  | | 6.52 ^[1]^ |  | |  |  |
| **bl** | 3-Me-2-Thi ^k^ | | 2,4-di-Me-Ph | | |  |  | |  | 6.59 ^[1]^  6.72 ^[39],l^ | | |  | |  |  | |  |  |
| **bm** | 3-Me-2-Thi ^k^ | | 2,6-di-Me-Ph | | |  |  | |  | 6.61 ^[1]^ | | |  | |  |  | |  |  |
| **bn** | 5-Me-2-Thi ^k^ | | 5-Me-2-Thi ^k^ | | |  |  | |  | 4.30 ^[40],e^ | | |  | |  |  | |  |  |
| **bo** | 2-Et-Ph | | 2-F-Ph | | |  |  | |  |  | | | 7.42 ^[37]^ | |  |  | |  |  |
| **bp** | 2-Cl-Ph | | 2-F-Ph | | |  |  | |  |  | | | 7.74 ^[37]^ | |  |  | |  |  |
| **bq** | 2-Br-Ph | | 2-F-Ph | | |  |  | |  |  | | | 7.66 ^[37]^ | |  |  | |  |  |
| **br** | 2-OCF_3_-Ph | | 2-F-Ph | | |  |  | |  |  | | | 7.28 ^[37]^ | |  |  | |  |  |
| **bs** | 2-OMe-Ph | | 2-F-Ph | | |  |  | |  |  | | | 7.27 ^[37]^ | |  |  | |  |  |
| **bt** | 2-Me-4-F-Ph | | 2-F-Ph | | |  |  | |  |  | | | 7.72 ^[37]^ | |  |  | |  |  |
| ^a^ Values are obtained for rat forebrain synaptosomes, unless noted otherwise. ^b^ Values obtained for neurons cultured from cerebral cortices of mouse embryos. ^c^ Values are obtained using [^3^H]-Tiagabine radioligand Binding Assays based on HEK293 cells stably expressing hGAT1, unless noted otherwise. ^d^ Values are obtained using MS Binding Assays with NNC-711 as MS marker. ^e^ Values are obtained in uptake assays with mGAT1. ^f^ Value given for the reduced structure (i.e. with butynyl tail). ^g^ Percentage inhibition at given concentration. ^h^ Value is obtained for rat hippocampal slices. ^i^ SKF-89976A. ^j^ SKF-100330A. ^k^ 2-Thi = Thien-2-yl. ^l^ Value for mixed isomers (*E/Z*). ^m^ 2-Pyr = Pyrrol-2-yl. ^n^ Tiagabine. | | | | | | | | | | | | | | | | | | | |
| ***Table S5 (continued):*** *SAR of GAT1 inhibitors with an alkene linker* | | | | | | | | | | | | | | | | | | | |
|  |  | |  | | | **5a** | **5b** | | | **5c** | | | | | **5d** | | | **5e** | |
|  |  | |  | | |  |  | | |  | | | | |  | | |  | |
| **Entry** | **R_1_** | | **R_2_** | | | **pIC_50_^a^** | **pIC_50_ ^a^** | | **pK_i_^b^** | **pIC_50_ ^a^** | | | **pK_i_^c^** | | **pIC_50_ ^a^** | **pK_i_^d^** | | **pIC_50_ ^a^** | **pK_i_^d^** |
| **bu** | 2-CF_3_-Ph | | 2-F-Ph | | |  |  | |  |  | | | 7.41 ^[37]^ | |  |  | |  |  |
| **bv** | 4-OMe-Ph | | 4-OMe-Ph | | |  |  | |  | 5.16 ^[40],e^ | | |  | |  |  | |  |  |
| **bw** | 4-F-Ph | | 4-F-Ph | | |  |  | |  | 5.70 ^[35]^ | | |  | |  |  | |  |  |
| **bx** | 4-Cl-Ph | | 4-Cl-Ph | | |  |  | |  | 6.66 ^[35]^ | | |  | |  |  | |  |  |
| **by** | 2-Me-4-Cl-Ph | | 1-Me-2-Pyr^m^ | | |  |  | |  | 6.83 ^[39],l^ | | |  | |  |  | |  |  |
| **bz** | Fluorenyl | | | | |  |  | |  | 5.72 ^[1]^, 5.70 ^[5]^ | | |  | |  |  | |  |  |
| **ca** |  | | | | |  | 4.87 ^[5]^  60-80 % at 1 μM ^[41],g^ | |  |  | | |  | |  |  | |  |  |
| **cb** |  | | | | |  | 60-80 % at 1 μM ^[41],g^ | |  |  | | |  | |  |  | |  |  |
| ^a^ Values are obtained for rat forebrain synaptosomes, unless noted otherwise. ^b^ Values obtained for neurons cultured from cerebral cortices of mouse embryos. ^c^ Values are obtained using [^3^H]-Tiagabine radioligand Binding Assays based on HEK293 cells stably expressing hGAT1, unless noted otherwise. ^d^ Values are obtained using MS Binding Assays with NNC-711 as MS marker. ^e^ Values are obtained in uptake assays with mGAT1. ^f^ Value given for the reduced structure (i.e. with butynyl tail). ^g^ Percentage inhibition at given concentration. ^h^ Value is obtained for rat hippocampal slices. ^i^ SKF-89976A. ^j^ SKF-100330A. ^k^ 2-Thi = Thien-2-yl. ^l^ Value for mixed isomers (*E/Z*). ^m^ 2-Pyr = Pyrrol-2-yl. ^n^ Tiagabine. | | | | | | | | | | | | | | | | | | | |

| ***Table S5 (continued):*** *SAR of GAT1 inhibitors with an alkene linker* | | | | | | | | | | | | |
| --- | --- | --- | --- | --- | --- | --- | --- | --- | --- | --- | --- | --- |
|  |  |  | | **5a** | **5b** | | **5c** | | **5d** | | **5e** | |
|  |  |  | |  |  | |  | |  | |  | |
| **Entry** | **R_1_** | **R_2_** | | **pIC_50_^a^** | **pIC_50_ ^a^** | **pK_i_^b^** | **pIC_50_ ^a^** | **pK_i_^c^** | **pIC_50_ ^a^** | **pK_i_^d^** | **pIC_50_ ^a^** | **pK_i_^d^** |
| **cc** |  | | |  | 60-80 % at 1 μM ^[41],g^ |  |  |  |  |  |  |  |
| **cd** |  | | |  | 60-80 % at 1 μM ^[41],g^ |  |  |  |  |  |  |  |
| **ce** |  | | H |  |  |  |  |  |  |  | 4.53 ^[42],e^ | 4.91  ^[42]^ |
| ^a^ Values are obtained for rat forebrain synaptosomes, unless noted otherwise. ^b^ Values obtained for neurons cultured from cerebral cortices of mouse embryos. ^c^ Values are obtained using [^3^H]-Tiagabine radioligand Binding Assays based on HEK293 cells stably expressing hGAT1, unless noted otherwise. ^d^ Values are obtained using MS Binding Assays with NNC-711 as MS marker. ^e^ Values are obtained in uptake assays with mGAT1. ^f^ Value given for the reduced structure (i.e. with butynyl tail). ^g^ Percentage inhibition at given concentration. ^h^ Value is obtained for rat hippocampal slices. ^i^ SKF-89976A. ^j^ SKF-100330A. ^k^ 2-Thi = Thien-2-yl. ^l^ Value for mixed isomers (*E/Z*). ^m^ 2-Pyr = Pyrrol-2-yl. ^n^ Tiagabine. | | | | | | | | | | | | |

| ***Table S6:*** *SAR of GAT1 inhibitors with an alkene and allene linker* | | | | | | | | | | | |
| --- | --- | --- | --- | --- | --- | --- | --- | --- | --- | --- | --- |
|  |  | **6a** | | **6b** | | **6c** | | **6d** | | **6e** | |
|  |  |  | |  | |  | |  | |  | |
| **Entry** | **R_1_** | **pIC_50_ ^a^** | **pK_i_^b^** | **pIC_50_ ^a^** | **pK_i_^b^** | **pIC_50_ ^a^** | **pK_i_^b^** | **pIC_50_ ^a^** | **pK_i_^b^** | **pIC_50_ ^a^** | **pK_i_^b^** |
| **a** | H |  |  |  |  |  |  | 3.89 ^[43]^ | 3.58 ^[43]^ |  |  |
| **b** | Ph | 6.79 ^[44]^  5.66 ^[44],c^  5.75 ^[44],d^ | 7.15 ^[44]^  6.09 ^[44],c^  4.94 ^[44],d^ | 7.27 ^[44]^ | 7.83 ^[44]^ |  |  | 4.73 ^[43]^ | 5.27 ^[43]^ | 5.42 ^[43]^  4.85 ^[43],e^  4.99 ^[43],f^ | 5.97 ^[43]^  5.88 ^[43],e^  5.10 ^[43],f^ |
| **c** | 2-F-Ph | 6.73 ^[44]^ | 7.57 ^[44]^ |  |  | 5.48 ^[24]^ | 6.19 ^[24]^ | 5.18 ^[43]^ | 5.41 ^[43]^ |  |  |
| **d** | 2-Cl-Ph | 7.34 ^[44]^ | 7.61 ^[44]^ | 6.39 ^[44]^ | 7.26 ^[44]^ | 5.45 ^[24]^ | 6.08 ^[24]^ | 5.71 ^[43]^ | 6.48 ^[43]^ |  |  |
| **e** | 3-Cl-Ph | 5.95 ^[44]^ | 6.43 ^[44]^ |  |  |  |  |  |  |  |  |
| **f** | 2-Br-Ph |  |  |  |  |  |  | 5.45 ^[43]^ | 6.20 ^[43]^ |  |  |
| **g** | 2-CF_3_-Ph |  |  |  |  |  |  | 5.32 ^[43]^ | 5.98 ^[43]^ |  |  |
| **h** | 2-OMe-Ph |  |  |  |  |  |  | 4.59 ^[43]^ | 5.51 ^[43]^ |  |  |
| **i** | 3-OMe-Ph |  |  |  |  |  |  | 4.86 ^[43]^ | 5.41 ^[43]^ |  |  |
| **j** | 2-isopropyl-Ph |  |  |  |  |  |  |  |  | 6.28 ^[43]^ | 6.51 ^[43]^ |
| **k** | 2-Ph-Ph |  |  |  |  |  |  |  |  | 6.48 ^[43]^  6.78 ^[43],g^  6.46 ^[43],h^ | 6.67 ^[43]^  7.10 ^[43],g^  7.07 ^[43],h^ |
| **l** | 2-Cl-4-F-Ph |  |  |  |  | 5.40 ^[24]^ | 6.51 ^[24]^ |  |  |  |  |
| **m** | 2,4-di-F-Ph | 6.60 ^[44]^ | 7.31 ^[44]^ | 6.02 ^[44]^ | 7.00 ^[44]^ | 5.55 ^[24]^ | 6.23 ^[24]^ |  |  | 5.55 ^[43]^  6.43 ^[43],h^ | 6.32 ^[43]^  6.63 ^[43],h^ |
| ^a^ Values are obtained in uptake assays with an assay system based on HEK293 cells stably expressing mGAT1. ^b^ Values are obtained using MS Binding Assays with NNC-711 as MS marker. ^c^ Value for Z-isomer. ^d^ reduced derivative (i.e. with butyl tail). ^e^ Value for meta derivative. ^f^ Value for para derivative. ^g^ Value for an 81:19 *(R)*:*(S)*-mixture (at the nipecotic acid). ^h^ Value for guvacine derivative. ^i^ Value for the *(R)*-nipecotic acid derivative. ^j^ 2-Thi = Thien-2-yl. | | | | | | | | | | | |
| ***Table S6:*** *SAR of GAT1 inhibitors with an alkene and allene linker* | | | | | | | | | | | |
|  |  | **6a** | | **6b** | | **6c** | | **6d** | | **6e** | |
|  |  |  | |  | |  | |  | |  | |
| **Entry** | **R_1_** | **pIC_50_ ^a^** | **pK_i_^b^** | **pIC_50_ ^a^** | **pK_i_^b^** | **pIC_50_ ^a^** | **pK_i_^b^** | **pIC_50_ ^a^** | **pK_i_^b^** | **pIC_50_ ^a^** | **pK_i_^b^** |
| **n** | 2,4-di-Cl-Ph | 7.28 ^[44]^  7.43 ^[44],i^ | 8.05 ^[44]^  8.33 ^[44],i^ | 5.83 ^[44]^ | 6.66 ^[44]^ | 6.11 ^[24]^ | 6.93 ^[24]^ |  |  | 6.19 ^[43]^  6.39 ^[43],h^ | 6.56 ^[43]^  7.09 ^[43],h^ |
| **o** | 2,6-di-Cl-Ph |  |  |  |  |  |  | 5.82 ^[43]^ | 6.31 ^[43]^ |  |  |
| **p** | 2-F-5-OMe-Ph | 5.91 ^[44]^ | 6.23 ^[44]^ |  |  |  |  |  |  |  |  |
| **q** | 2-Cl-5-OMe-Ph |  |  |  |  |  |  | 5.33 ^[43]^ | 6.07 ^[43]^ |  |  |
| **r** | 2,4-di-OMe-Ph |  |  |  |  |  |  |  |  | 4.83 ^[43]^ | 5.44 ^[43]^ |
| **s** | 2,5-di-OMe-Ph |  |  |  |  |  |  | 5.68 ^[43]^ | 6.34 ^[43]^ |  |  |
| **t** | 3,5-di-OMe-Ph |  |  |  |  |  |  | 5.37 ^[43]^ | 6.02 ^[43]^ |  |  |
| **u** | 2,4-di-CF_3_-Ph |  |  |  |  |  |  |  |  | 5.78 ^[43]^ | 6.19 ^[43]^ |
| **v** | 2,4-di-Me-Ph | 6.39 ^[44]^ | 6.66 ^[44]^ |  |  |  |  |  |  |  |  |
| **w** | 2,6-di-Me-Ph |  |  |  |  |  |  |  |  | 6.03 ^[43]^ | 6.17 ^[43]^ |
| **x** | 2,4,6-tri-Me-Ph | 6.27 ^[44]^ | 6.52 ^[44]^ |  |  |  |  |  |  |  |  |
| **y** | 2-Thi ^j^ |  |  |  |  | 5.39 ^[45]^ | 6.04 ^[45]^ |  |  |  |  |
| **z** | 3-Me-2-Thi ^j^ |  |  |  |  | 5.05 ^[45]^ | 5.89 ^[45]^ |  |  |  |  |
| **aa** | Naphthyl |  |  |  |  |  |  | 5.22 ^[43]^ | 5.95 ^[43]^ |  |  |
| **ab** | CH_2_-Ph | 5.72 ^[44]^ | 6.16 ^[44]^ |  |  |  |  |  |  |  |  |
| **ac** | C(O)Ph | 4.86 ^[44]^ | 5.72 ^[44]^ |  |  |  |  |  |  |  |  |

^a^ Values are obtained in uptake assays with an assay system based on HEK293 cells stably expressing mGAT1. ^b^ Values are obtained using MS Binding Assays with NNC-711 as MS marker. ^c^ Value for Z-isomer. ^d^ reduced derivative (i.e. with butyl tail). ^e^ Value for meta derivative. ^f^ Value for para derivative. ^g^ Value for an 81:19 *(R)*:*(S)*-mixture (at the nipecotic acid). ^h^ Value for guvacine derivative. ^i^ Value for the *(R)*-nipecotic acid derivative. ^j^ 2-Thi = Thien-2-yl.

| ***Table S7:*** *SAR of GAT1 inhibitors with an alkyne linker* | | | | | | | | | |  |
| --- | --- | --- | --- | --- | --- | --- | --- | --- | --- | --- |
|  |  | **7a** | | **7b** | | **7c** | | **7d** | |  |
|  |  |  | |  | |  | |  | |  |
| **Entry** | **R_1_** | **pIC_50_^a^** | **pK_i_^b^** | **pIC_50_ ^a^** | **pK_i_^b^** | **pIC_50_ ^a^** | **pK_i_^b^** | **pIC_50_ ^a^** | **pK_i_^b^** | |
| **a** | Ph | 7.00 ^[46]^ | 7.61 ^[46]^ |  |  | 5.42 ^[46]^ | 6.16 ^[46]^ |  |  | |
| **b** | 2-Cl-Ph | 7.29 ^[46]^ | 7.91 ^[46]^ |  |  | 5.80 ^[46]^ | 6.29 ^[46]^ |  |  | |
| **c** | 2-F-Ph | 7.12 ^[46]^ | 7.77 ^[46]^ |  |  | 5.56 ^[46]^ | 6.29 ^[46]^ |  |  | |
| **d** | 2-Me-Ph | 7.22 ^[46]^ | 8.13 ^[46]^ | 7.60 ^[46]^ | 8.32 ^[46]^ | 5.89 ^[46]^ | 6.27 ^[46]^ |  |  | |
| **e** | 4-Cl-Ph | 6.73 ^[46]^ | 7.37 ^[46]^ |  |  | 5.47 ^[46]^ | 6.07 ^[46]^ |  |  | |
| **f** | 4-OMe-Ph |  |  |  |  |  |  | 5.43 ^[15]^ | 5.96 ^[15]^ | |
| **g** | 2,4-di-Cl-Ph | 7.26 ^[46]^ | 8.16 ^[46]^ | 7.72 ^[46]^ | 8.31 ^[46]^ | 6.03 ^[46]^ | 6.55 ^[46]^ |  |  | |
| **h** | 2,4-di-F-Ph | 7.31 ^[46]^ | 8.02 ^[46]^ | 7.56 ^[46]^ | 8.21 ^[46]^ | 5.70 ^[46]^ | 6.42 ^[46]^ |  |  | |
| **i** | 2,4-di-Me-Ph | 6.68 ^[46]^ | 7.68 ^[46]^ |  |  | 5.46 ^[46]^ | 6.09 ^[46]^ |  |  | |
| **j** | 2-Cl-4-F-Ph | 7.35 ^[46]^ | 8.13 ^[46]^ | 7.68 ^[46]^ | 8.33 ^[46]^ | 5.77 ^[46]^ | 6.46 ^[46]^ |  |  | |
| **k** | 2-Me-4-Cl-Ph | 6.80 ^[46]^ | 7.78 ^[46]^ |  |  | 6.01 ^[46]^ | 6.40 ^[46]^ |  |  | |
| **l** | 2-Me-4-F-Ph | 7.11 ^[46]^ | 7.90 ^[46]^ |  |  | 5.88 ^[46]^ | 6.18 ^[46]^ |  |  | |
| **m** | CH_2_-Ph | 5.87 ^[46]^ | 6.49 ^[46]^ |  |  | 5.13 ^[46]^ | 5.66 ^[46]^ |  |  | |
| ^a^ Values are obtained in uptake assays with an assay system based on HEK293 cells stably expressing mGAT1. ^b^ Values are obtained using MS Binding Assays with NNC-711 as MS marker. | | | | | | | | | |  |

**References:**

1. Andersen KE, Braestrup C, Groenwald FC, Joergensen AS, Nielsen EB, Sonnewald U, et al. The synthesis of novel GABA uptake inhibitors. 1. Elucidation of the structure-activity studies leading to the choice of (R)-1-[4,4-bis(3-methyl-2-thienyl)-3-butenyl]-3-piperidinecarboxylic acid (Tiagabine) as an anticonvulsant drug candidate. J Med Chem. 1993;36:1716-25. <https://10.1021/jm00064a005>.

2. Falch E, Korgsgaard-Larsen P. GABA uptake inhibitors. Syntheses and structure—activity studies on GABA analogues containing diarylbutenyl and diarylmethoxyalkyl N-substituents. Eur J Med Chem. 1991;26:69-77. <https://10.1016/0223-5234(91)90214-8>.

3. Pavia MR, Lobbestael SJ, Nugiel D, Mayhugh DR, Gregor VE, Taylor CP, et al. Structure-activity studies on benzhydrol-containing nipecotic acid and guvacine derivatives as potent, orally-active inhibitors of GABA uptake. J Med Chem. 1992;35:4238-48. <https://10.1021/jm00100a032>.

4. Pavia MR. Various N-substituted 3-piperidine carboxylic acids or N-substituted 3-pyridinecarboxylic acids and derivatives thereof US4772615A; 1988.

5. Andersen KE, Sørensen JL, Lau J, Lundt BF, Petersen H, Huusfeldt PO, et al. Synthesis of novel γ-aminobutyric acid (GABA) uptake inhibitors. 5. Preparation and structure−activity studies of tricyclic analogues of known GABA uptake inhibitors. J Med Chem. 2001;44:2152-63. <https://10.1021/jm990513k>.

6. Sørensen PO, Lau J, Andersen Knud E, Petersen H, Lundt BF. N-Substituted azaheterocyclic carboxylic acids and esters thereof. WO9500485A1; 1995.

7. Knudsen LJS, Jørgensen AS, Andersen KE, Sonnewald U. N-Substituted Azaheterocyclic Carboxyclic Acids and Pharmaceutical Uses. US5071859; 1991.

8. Andersen KE, Lau J, Lundt BF, Petersen H, Huusfeldt PO, Suzdak PD, et al. Synthesis of novel GABA uptake inhibitors. Part 6: Preparation and evaluation of N-Ω asymmetrically substituted nipecotic acid derivatives. Falch E, Krogsgaard-Larsen P. GABA uptake inhibitors containing mono- and diarylmethoxyalkyl N-substituents. Drug Des Deliv. 1989;4:205-15. 2001;9:2773-85. <https://10.1016/S0968-0896(01)00148-1>.

9. Petersen H, Andersen KE, Sørensen PO, Lau J, Lundt BF. Heterocyclic chemistry. US5604242; 1997.

10. Andersen KE, Sorensen JL, Huusfeldt PO, Knutsen LJ, Lau J, Lundt BF, et al. Synthesis of novel GABA uptake inhibitors. 4. Bioisosteric transformation and successive optimization of known GABA uptake inhibitors leading to a series of potent anticonvulsant drug candidates. J Med Chem. 1999;42:4281-91. <https://10.1021/jm980492e>.

11. Andersen KE, Knudsen LJS, Sonnewald U, Sørensen PO. Heterocyclic carboxylic acids. US5198451; 1993.

12. Falch E, Krogsgaard-Larsen P. GABA uptake inhibitors containing mono- and diarylmethoxyalkyl N-substituents. Drug Des Deliv. 1989;4:205-15.

13. Dhar TGM, Borden LA, Tyagarajan S, Smith KE, Branchek TA, Weinshank RL, et al. Design, synthesis and evaluation of substituted triarylnipecotic acid derivatives as GABA uptake inhibitors: identification of a ligand with moderate affinity and selectivity for the cloned human GABA transporter GAT-3. J Med Chem. 1994;37:2334-42. <https://10.1021/jm00041a012>.

14. Pabel J, Faust M, Prehn C, Wörlein B, Allmendinger L, Höfner G, et al. Development of an (S)-1-{2-[Tris(4-methoxyphenyl)methoxy]ethyl}piperidine-3-carboxylic acid [(S)-SNAP-5114] carba analogue inhibitor for murine γ-aminobutyric acid transporter type 4. ChemMedChem. 2012;7:1245-55. <https://10.1002/cmdc.201200126>.

15. Tóth K, Höfner G, Wanner KT. Synthesis and biological evaluation of novel N-substituted nipecotic acid derivatives with an alkyne spacer as GABA uptake inhibitors. Bioorg Med Chem. 2018;26:3668-87. <https://10.1016/j.bmc.2018.05.049>.

16. Tóth K, Höfner G, Wanner KT. Synthesis and biological evaluation of novel N-substituted nipecotic acid derivatives with a cis-alkene spacer as GABA uptake inhibitors. Bioorg Med Chem. 2019;27:822-31. <https://10.1016/j.bmc.2019.01.024>

17. Tóth K, Höfner G, Wanner KT. Synthesis and biological evaluation of novel N-substituted nipecotic acid derivatives with a trans-alkene spacer as potent GABA uptake inhibitors. Bioorg Med Chem. 2018;26:5944-61. <https://10.1016/j.bmc.2018.11.002>.

18. Quandt G, Höfner G, Pabel J, Dine J, Eder M, Wanner KT. First photoswitchable neurotransmitter transporter inhibitor: light-induced control of γ-aminobutyric acid transporter 1 (GAT1) activity in mouse brain. J Med Chem. 2014;57:6809-21. <https://10.1021/jm5008566>.

19. Lutz T, Wein T, Höfner G, Pabel J, Eder M, Dine J, et al. Development of new photoswitchable azobenzene based γ-aminobutyric acid (GABA) uptake inhibitors with distinctly enhanced potency upon photoactivation. J Med Chem. 2018;61:6211-35. <https://10.1021/acs.jmedchem.8b00629>.

20. Knutsen LJS, Andersen KE, Lau J, Lundt BF, Henry RF, Morton HE, et al. Synthesis of novel GABA uptake inhibitors. 3. Diaryloxime and diarylvinyl ether derivatives of nipecotic acid and guvacine as anticonvulsant agents. J Med Chem. 1999;42:3447-62. <https://10.1021/jm981027k>.

21. Quandt G, Höfner G, Wanner KT. Synthesis and evaluation of N-substituted nipecotic acid derivatives with an unsymmetrical bis-aromatic residue attached to a vinyl ether spacer as potential GABA uptake inhibitors. Bioorg Med Chem. 2013;21:3363-78. <https://10.1016/j.bmc.2013.02.056>.

22. Andersen KE, Knutsen LJS, Sorensen PO, Lundt BF, Lau J, Petersen H. N-substituted azaheterocyclic carboxylic acids. US5348965; 1994.

23. Knutsen LJS, Andersen KE, Jorgensen AS, Sonnewald U. Azacyclic carboxylic acid derivatives, their preparation and use. EP0342635A1; 1989.

24. Sindelar M, Lutz TA, Petrera M, Wanner KT. Focused pseudostatic hydrazone libraries screened by mass spectrometry binding assay: optimizing affinities toward γ-aminobutyric acid transporter 1. J Med Chem. 2013;56:1323-40. <https://10.1021/jm301800j>.

25. Sindelar M, Wanner KT. Library screening by means of mass spectrometry (MS) binding assays—exemplarily demonstrated for a pseudostatic library addressing γ-aminobutyric acid (GABA) transporter 1 (GAT1). ChemMedChem. 2012;7:1678-90. <https://10.1002/cmdc.201200201>.

26. Kern FT, Wanner KT. Generation and screening of oxime libraries addressing the neuronal GABA transporter GAT1. ChemMedChem. 2015;10:396-410. <https://10.1002/cmdc.201402376>.

27. Gabriel J, Höfner G, Wanner KT. Combination of MS binding assays and affinity selection mass spectrometry for screening of structurally homogenous libraries as exemplified for a focused oxime library addressing the neuronal GABA transporter 1. Eur J Med Chem. 2020;206:112598. <https://10.1016/j.ejmech.2020.112598>.

28. Kern F, Wanner KT. Screening oxime libraries by means of mass spectrometry (MS) binding assays: Identification of new highly potent inhibitors to optimized inhibitors γ-aminobutyric acid transporter 1. Bioorg Med Chem. 2019;27:1232-45. <https://10.1016/j.bmc.2019.02.015>.

29. Petersen H, Andersen KE, Sørensen PO, Lau J, Petersen HB, Lundt BF. 1-Substituted, 3-carboxylic acid piperidine derivatives. US5608069; 1997.

30. Petersen H, Andersen KE, Sørensen PO, Lau J, Lundt BF. N-substituted azaheterocyclic carboxylic acids and esters thereof. US5639766; 1997.

31. Rudy H-KA, Höfner G, Wanner KT. Synthesis and biological evaluation of novel N-substituted nipecotic acid derivatives with tricyclic cage structures in the lipophilic domain as GABA uptake inhibitors. Med Chem Res. 2021;30:586-609. <https://10.1007/s00044-020-02647-9>.

32. Wein T, Petrera M, Allmendinger L, Höfner G, Pabel J, Wanner KT. different binding modes of small and large binders of GAT1. ChemMedChem. 2016;11:509-18. <https://10.1002/cmdc.201500534>.

33. Ali FE, Bondinell WE, Dandridge PA, Frazee JS, Garvey E, Girard GR, et al. Orally active and potent inhibitors of γ-aminobutyric acid uptake. J Med Chem. 1985;28:653-60. <https://10.1021/jm50001a020>.

34. Bondinell WE, Lafferty JJ, Zirkle CL. Inhibition of GABA uptake by N-substituted azaheterocyclic carboxylic acids and their esters. US4383999A; 1983.

35. Yunger LM, Fowler PJ, Zarevics P, Setler PE. Novel inhibitors of gamma-aminobutyric acid (GABA) uptake: anticonvulsant actions in rats and mice. J Pharmacol Exp Ther. 1984;228:109-15.

36. Larsson OM, Falch E, Krogsgaard-Larsen P, Schousboe A. Kinetic characterization of inhibition of γ-aminobutyric acid uptake into cultured neurons and astrocytes by 4,4-diphenyl-3-butenyl derivatives of nipecotic acid and guvacine. J Neurochem. 1988;50:818-23. <https://10.1111/j.1471-4159.1988.tb02986.x>.

37. Pizzi DA, Leslie CP, Fabio RD, Seri C, Bernasconi G, Squaglia M, et al. Stereospecific synthesis and structure–activity relationships of unsymmetrical 4,4-diphenylbut-3-enyl derivatives of nipecotic acid as GAT-1 inhibitors. Bioorg Med Chem Lett. 2011;21:602-5. <https://10.1016/j.bmcl.2010.09.025>.

38. Grønvald FC, Braestrup C. Amino acid derivatives. WO8700171; 1987.

39. Sonnewald U. Amino acid derivatives. US4931450A; 1990.

40. Zhang J-g, Jiang C-s, Zheng J-b, Wen R, Lin G-q. A new synthesis method and GABA transporters inhibitory activities of tiagabine and its analogues. Chem Res Chin Univ. 2006;22:351-5. <https://10.1016/S1005-9040(06)60114-1>.

41. Sindelar K, Silhankova A, Urban J, Metys J, Valchar M, Polivka Z. Antihistamine substances. Tricyclic analogues of N-(4,4-diphenyl-3-butene-1-yl)nipecotic acid and some related compounds. Collect Czechoslov Chem Comm. 1994;59:667-74. <https://10.1135/cccc19940667>.

42. Daerr M, Allmendinger L, Höfner G, Wanner KT. Synthesis and biological evaluation of fluorescent GAT-ligands based on asymmetric substituted BODIPY dyes. Med Chem Res. 2020;29:767-82. <https://10.1007/s00044-020-02521-8>.

43. Schaarschmidt M, Höfner G, Wanner KT. Synthesis and biological evaluation of nipecotic acid and guvacine derived 1,3-disubstituted allenes as inhibitors of murine GABA transporter mGAT1. ChemMedChem. 2019;14:1135-51. <https://10.1002/cmdc.201900170>.

44. Petrera M, Wein T, Allmendinger L, Sindelar M, Pabel J, Höfner G, et al. development of highly potent GAT1 inhibitors: synthesis of nipecotic acid derivatives by suzuki–miyaura cross-coupling reactions. ChemMedChem. 2016;11:519-38. <https://10.1002/cmdc.201500490>.

45. Lutz T. Syntheses of nipecotic acid derivatives with new N-arylalkenyl and N-arylalkynyl substituents and with new photoswitchable residues as mGAT1 inhibitors [PhD Thesis]. München: Ludwig-Maximilians-Universität München; 2017.

46. Lutz T, Wein T, Höfner G, Wanner KT. Development of highly potent GAT1 inhibitors: synthesis of nipecotic acid derivatives with N‐arylalkynyl substituents. ChemMedChem. 2017;12:362-71. <https://10.1002/cmdc.201600599>.
